# Games Used With Serious Purposes: A Systematic Review of Interventions in Patients With Cerebral Palsy

**DOI:** 10.3389/fpsyg.2018.01712

**Published:** 2018-09-19

**Authors:** Sílvia Lopes, Paula Magalhães, Armanda Pereira, Juliana Martins, Carla Magalhães, Elisa Chaleta, Pedro Rosário

**Affiliations:** ^1^School of Psychology, University of Minho, Braga, Portugal; ^2^Associação de Paralisia Cerebral de Braga, Braga, Portugal; ^3^School of Psychology and Science Social, University of Évora, Évora, Portugal

**Keywords:** cerebral palsy, serious games, videogames, rehabilitation, systematic review

## Abstract

The purpose of the present systematic review was to examine extant research regarding the role of games used seriously in interventions with individuals with cerebral palsy. Therefore, PubMed, PsyINFO, Web of Science, Scopus, and IEEE databases were used. Search terms included: “serious games” OR “online games” OR “video games” OR “videogame” OR “game based” OR “game” AND “intervention” AND “cerebral palsy.” After the full reading and quality assessment of the papers, 16 studies met the inclusion criteria. The majority of the studies reported high levels of compliance, motivation, and engagement with game-based interventions both at home and at the clinical setting intervention. Regarding the effectiveness of the use of games, the results of the studies show both positive and negative results regarding their effectiveness. The efficacy was reported to motor function (i.e., improvements in the arm function, hand coordination, functional mobility, balance and gait function, postural control, upper-limbs function) and physical activity. Findings of this review suggest that games are used as a complement to conventional therapies and not as a substitute. Practitioners often struggle to get their patients to complete the assigned homework tasks, as patients display low motivation to engage in prescribed exercises. Data of this review indicates the use of games as tools suited to promote patients’ engagement in the therapy and potentiate therapeutic gains.

## Introduction

Cerebral palsy (CP) refers to a group of permanent, non-progressive, developmental disorders that mainly affects movement and posture (Bax et al., 2005), with associated secondary impairments such as cognitive, language, and visual impairments (Odding et al., 2006), and with repercussions in daily activity. CP is considered the most common cause of childhood physical disability (Novak et al., 2013), with an estimated prevalence of 1.5–2.5 children per 1000 live births (Surveillance of Cerebral Palsy in Europe, 2002). Importantly, CP is a permanent neurologic, non-progressive, and lifelong condition. CP can be divided into two major categories considering the body topography of the lesion: bilateral and unilateral (Rosenbaum et al., 2007; Graham et al., 2016). In bilateral CP, two sides of the body could be totally or partially impaired (e.g., diplegia), while unilateral CP is characterized by only one part of the body being compromised (e.g., hemiplegia). Another important clinical classification is based on the muscle tonus. The four types include dyskinetic, ataxic, spastic, and mixed (Straub and Obrzut, 2009).

The severity of this clinical picture is wide and has different levels of impact on the daily life of youth with CP’s (e.g., struggle to succeed in school; struggle in their social relationships; reduce employment opportunities). Hence, the main goals of intervention ought to promote the autonomy and well-being of youth with CP through the appropriate combination of interventions tailored to the specificity of this condition (Rosenbaum, 2003). For these reasons, CP interventions need to consider a neurodevelopmental approach. The goal of this approach is to enhance the children’s control over the following somatic and mental functions: sensorimotor components (Krigger, 2006), postural control (e.g., de Graaf-Peters et al., 2007), sensation (e.g., Hoon, 2005), perception (e.g., Auld et al., 2012), memory (e.g., Gehring et al., 2011), attention (e.g., Moore Sohlberg et al., 2000), and executive function (e.g., Levine et al., 2000; Stadskleiv et al., 2016). Additionally, the systematic review by Novak et al. (2013) concluded that effective interventions with children with CP focused on: tasks to improve motor function of ankle and hands, overall fitness training, and management of specific health conditions (i.e., hip status, spasticity, ulcers, bone density, and convulsions). Most importantly, these authors highlighted the importance of context focused therapy and goal-directed training, home-based therapies, and occupational therapies (e.g., activities of daily living) to improve the clinical picture of the patients.

Altogether, these aspects provide an important framework to guide treatment for these patients. Still, it is important to underline that CP is a mutable disorder (CPF, 2017). That is, with proper stimulation CP’s functionality profile can be improved.

In an attempt to further improve the care and rehabilitation of patients with CP, new approaches have been applied recently, specifically the use of games within the interventions’ schemes (e.g., Tatla et al., 2013; Lawrence et al., 2016; Cooper and Williams, 2017; Ravi et al., 2017). Due to their design elements, games can offer rewarding and engaging experiences that can be shared with other players in the form, for instance, of “achievements” (e.g., number of points, ranking) (see Boyle et al., 2012). In fact, the interactive nature of the games enables constructive, situational, and experiential learning opportunities, enhanced by active game experimentation (Squire, 2008; Hainey et al., 2011; Girard et al., 2012). For these reasons, games have been employed as a way of promoting the engagement and participation of patients with CP in their rehabilitation programs (e.g., Tatla et al., 2013; Bonnechère et al., 2014a; Lawrence et al., 2016; Cooper and Williams, 2017; Ravi et al., 2017).

Games are as old as human civilization itself. For centuries, they have been used to entertain both children and adults. In today’s world, games and videogames are ubiquitous in a plethora of environments.

In fact, the growth of digital games at the beginning of the 21st century has led to an increase in interest in their potential to promote some transversal skills (Green and Bavelier, 2006). For example, some authors have found that playing video games (i.e., games played on computers or on games platforms – e.g., Nintendo, Sony PlayStation) can improve attention, memory, and overall performance in the executive functions (e.g., Boot et al., 2008; Colzato et al., 2012). However, caution may be needed when interpreting these results as these studies compared the performance of gamers vs. non-gamers (Bavelier et al., 2012; see also Simons et al., 2016). Nevertheless, these results bring some enthusiasm about the potential of entertainment games as an attractive and fun way to train and improve useful skills (Connolly et al., 2012). Thus, it comes as no surprise that there is an emergence of a new category of games that can be used as tools of enhancement: serious games (SG). SG may be defined as “a mental contest, played with a computer in accordance with specific rules, that uses entertainment to further government or corporate training, education, health, public policy, and strategic communication objectives” (Zyda, 2005, p. 26). Thus, SG have specific goals and are developed for learning, teaching, training, informing, and rehabilitating purposes (Susi et al., 2007; Moyà Alcover et al., 2011). Moreover, SG allow for the augmentation of training skills by favoring attitude change and openness to the transmission of knowledge (Michael and Chen, 2006). These games can be used in different contexts and domains, such as the military, government, education, healthcare, or corporate settings (Susi et al., 2007) to promote the cognitive and affective dimensions (O’Neil et al., 2005). The growing interest in the study of SG helps explain the need to conduct meta-analyses and systematic reviews to look at their efficacy in enhancing some aspect of the individual. Particularly, these reviews have focused on how SG have been used in interventions within the last decade. For example, authors have looked at SG for health and education (Bartolomé et al., 2011), with reported social (e.g., user’s motivation) and medical (e.g., assessment and measurement of progress) benefits for the participants involved in such interventions. Other reviews have looked at the potential of SG for learning (e.g., Boyle et al., 2016) with results highlighting their role in knowledge acquisition in several topics, such as adolescent sexual relationship, nutrition, English as a second language.

Serious games are games designed to convey learning and promote specific skills (e.g., Gros, 2007; Parong et al., 2017). However, literature has been reporting the use of commercial-off-the-shelf (COTS) games to improve generic or transversal skills or for rehabilitation purposes (e.g., Van Eck, 2006; Crosbie et al., 2007; Laver et al., 2011; Wuang et al., 2011; Levac et al., 2012; Lucas et al., 2016; Mat Rosly et al., 2016).

In fact, despite the implicit entertaining nature and commercial intents of games (e.g., COTS) they may be used with serious purposes (Susi et al., 2007; Backlund and Hendrix, 2013). This practice stresses the limitation of a descriptor for games focused on their nature (e.g., entertaining vs. non-entertaining games), and unfolds the need to introduce a new concept focused on the purpose of using games. Moreover, this approach provides a rationale for including in the current review papers reporting the use of games with serious purposes independently of their nature.

Games used seriously (GUS) refers to games used with serious purposes, regardless of their nature (e.g., entertain *vs.* serious). This new concept draws investigators’ attention to the features of the COTS/entertainment games. Although these games are not designed with the purpose to support the acquisition and learning of specific competencies, they can help accomplish these competencies when played with serious purposes (Connolly et al., 2012). Therefore, GUS is rooted on the literature stressing the need to theoretically distinguish games used with serious purposes from those with entertaining purposes (e.g., Francesco and Paolis, 2014; Kitchen and Humphreys, 2014; Theng et al., 2015). This concept overcomes the limitations inherent to the distinction between non-entertainment nature *vs.* entertainment nature of the games, by focusing on the purpose of the use of the games, namely a serious purpose.

### Purpose

Considering the rationale above stated, the present review aims to deepen the understanding of the role GUS (e.g., serious games, video games, online games) may play in interventions with rehabilitation or therapeutic purposes involving patients with CP. Specifically, the main aims are to map the following characteristics of the interventions: the purpose (e.g., motor rehabilitation), the duration of the overall intervention and the sessions, the contexts in which the interventions took place, the interveners involved (e.g., patients, family), the nature of the game used, the measures used to evaluate the efficacy (e.g., interview), and the outcomes. We aim to answer questions such as: does the incorporation of GUS into interventions have the main purpose of motivating and engaging individuals in the process? Alternatively, does the inclusion of GUS have purposes other than motivating and engaging individuals (e.g., educational, rehabilitation, physical therapy, cognitive training)? Are GUS a complement to interventions or are they the core? How is the efficacy of these interventions being assessed?

## Methods

The present systematic review focused on understanding the role that GUS may play in interventions involving individuals with CP. Cochrane Collaboration (Higgins and Green, 2011) guidelines were followed in the development of the manuscript, including the search, the selection, and the extraction of the material as well as the guidelines of The Preferred Reporting Items for Systematic Reviews and Meta-Analysis (PRISMA) (Moher et al., 2009).

### Study Selection

Our research included articles published until April 2018; moreover, the following databases were used in the literature search: PsycINFO, Pubmed, IEEE, Scopus, and Web of Science. The keywords used for the search were “serious games,” “online games,” “video games,” “videogame,” “game based,” “game,” “intervention,” and “cerebral palsy” following this equation: (“serious games” OR “online games” OR “video games” OR “videogames” OR “game based” OR “game” AND “intervention” AND “cerebral palsy”). After the searches, the duplicates were removed and titles remaining were screened for reference to interventions with GUS. Afterward, abstracts of the remaining papers were screened for eligibility.

### Data Extraction

The reviewers followed the PRISMA (Moher et al., 2009) guidelines for the data abstraction. The PRISMA Statement provides an evidence-based 27-item checklist (e.g., on objectives, methodology, limitations) for systematic reviews and meta-analyses.

The team included SL, AP, PM, and JM. The first author (SL) screened the titles and abstracts. In turn, two researchers (SL and PM) independently conducted the eligibility phase. When discrepancies arose between SL and PM, a third researcher (AP) refereed the discussion (i.e., SL and PM reviewed all papers and, when any doubt arose regarding a paper, AP read the paper to later discuss it with the team). Additionally, SL and PM followed a set of inclusion and exclusion criteria (see below) for the inclusion through abstract screening (see **Appendix B**). Finally, information from the selected articles was organized as follows: (i) reference (authors, year of publication), (ii) sample information (sample size, clinical setting, and age), (iii) purpose of the intervention, (iv) serious game used (intervention, frequency), (v) measures (rating criteria, task, and assessment tools), and (vi) results and conclusion. Lastly, SL extracted all relevant information to the tables and JM independently read all the included papers and reviewed the extracted data (see **Table 1**).

**Table 1:** Summary of the studies included in the systematic review.

Reference	Design	Sample	Intervention setting	Intervention: Nature of game, frequency, type, and duration	Measures	Outcomes	Quality score
Howcroft et al. (2012a) (Canada)	Single-group, experimental study	15 children with CP [9.77 (SD) 1.78 years old]	Clinical setting Single study	AVG Game played 8 min twice (1 – against computer; 2 – against the member of research team) with a 5-min a rest in between	-Anthropometric measurements; -Energy measurements; -Motion capture; -Muscle activity measures; -Self-report measures of perceived exertion; -Physical activity enjoyment.	Active video games do elicit muscle activation and energy expenditure. Can be regarded as promoters of physical activity.	11
Preston et al. (2015) (United Kingdom)	Single-blind RCT	15 children with CP [9.2 (SD) 2.5 years old] 8 to the device group.	Home setting	Computer Games The computer-assisted arm rehabilitation gaming Technology: Played 30 min a day Duration: 6 weeks	-Activities questionnaire reported by parents (The ABILHAND-kids and The Canadian Occupational Performance Measures); - Family diaries (describe the rehabilitation process, questionnaire evaluating the use of the gaming system, engagement with games, and participation in the study.	The study failed to observe improvements in the arm function. Game-based training should be regarded as a complement of traditional rehabilitation sessions, particularly with the possibility of including competitive and collaborative play.	13
Howcroft et al. (2012b) (Canada)	Single-group, experimental study	17 children with CP [9.43 (SD) 1.51 years old]	Clinical setting Single study	AVG Each child played each game (4 games) in a randomized order for 8 min, on a preselected beginners level, and with a rest period of 5 min between each game. This time per game allowed children to engage in several (2–3) matches/rounds of each game with brief (10 s) intervals between each match.	-Anthropometric (Body mass); -Energy expenditure -Motion capture; -Muscle activity; - OMNI Scale of Perceived Exertion (OMNI); - Physical Activity Enjoyment Scale (PACES); -Video recorded.	AVGs have potential for neuromuscular reeducation, but are ineffective for building endurance or strength. Games can be strategically selected as a function of the goals of the therapy. A high level of enjoyment was reported (PACES).	10
Levac et al. (2017) (Canada)	Pilot non-randomized controlled trial	11 children with CP (range 7–8 years old)	Home setting	AVG -5 children received 1 h of VR training for 5 days followed by a 6-week home AVG program (supervised online by a physical therapist) -Six children completed only the 6-week home AVG program	- Postural responses (Computer Assisted Rehabilitation Environment); -Six Minute Walk Test; - Gross Motor Function Measure Challenge Module; - Participant perceptions of the AVG exercise program.	Study showed no differences between groups in terms of functional mobility after the 6-week training with AVG.	11
MacIntosh et al. (2017) (Canada)	Prospective repeated measures	10 children with CP [12.4 (SD) 1.8 years old]	Clinical setting	Exergames 30 min of exergaming was an activity on each of the 9 days. 6 exergaming sessions using each of the 3 algorithms	-Game success; -The percentage of playtime; -Intrinsic Motivation Inventory.	None singled-out the other in minimizing differences in game success, time above 40% heart rate reserve, and enjoyment as a function of participants’ Gross Motor Function level.	13
Ramstrand and Lygnegård (2012) (Sweden)	Randomized cross-over design	18 children with CP (age range 8-17)	Home setting	AVG 5 weeks of playing Wii Fit games (e.g., Soccer Heading, Ski Slalom) for a minimum of 30 min, 5 days a week.	- PRO Balance Master to test balance; -Delsys Bagnoli EMG system; - Standing balance (modified sensory organization test --MSot); - Reactive balance- Lateral weight shifting ability; - Diary to record their playing time.	No significant difference was observed between the three testing occasions for any of the balance measures investigated.	12
Liu et al. (2017) (China)	Repeated measures Case study	20 children with CP [8.7 (SD) 2.8 years old] 3 children with CP [10 (SD) 2 years old]	Home setting Single Study	AVG (1) All the subjects received about 30mins/visit of game intervention training in 1 month (2–3 visits per week); (2) once a week (about 30 min/visit) for one and a half months following the S1 stage. CP2 and CP3 had shorter training times in S2 to accommodate their school work.	-Fugl-Meyer Assessment Scale; -ADL Scale; -Subjects’ ability to eat, drink, wear cloths; - Self reported measures (comfort, understandability, ease, efficacy of feedback, interestingness, attention, and fatigue).	All the subjects yielded improved their game performance during the long-term training.	10
Jaume-i-Capo et al. (2014) (Spain)	Experimental study	9 adults with CP (age range of 27-57);	Clinical setting	Serious games 1 session per week, physiotherapy intervention program. Participants had to practice with serious game for balance rehabilitation therapy for at least 20 min. Duration: 24 weeks	- Functional Assessment of Balance; - A case report form (feelings about the game daily); -Fatigue (analog scale).	Significant functional improvement in balance and gait function scores resulting in indicators of greater independence for participating adults.	10
Burdea et al. (2013) (United States)	Case study	3 children with CP (age range 7–12)	Research setting	Computer games The two custom-made virtual reality games were played while participants were seated, and controlled with the foot being trained, that is, it was trained one ankle at-a-time for strength, motor control, and coordination, for 36 rehabilitation sessions (12 weeks, three times/week).	-Ankle strength; - Gross Motor Function Measure (GMFM); - Maximum ankle dorsiflexion at initial contact during gait and gait speed; - Quality of life (based on child and parent self-report - Pediatric Quality of Life Inventory (PedsQL]). - Game performance (game scores as reflection of ankle motor control and endurance).	Gait function improved substantially in ankle kinematics, speed, and endurance. Overall function (GMFM) indicated improvements that were typical of other ankle strength training programs. Quality of life (PedsQL) increased beyond what would be considered a minimal clinical important difference.	10
Diez Alegre and Cano de la Cuerda, 2012 (Spain)	A prospective evaluation pre-and post-intervention pilot study	10 patients with CP, [45.80 (SD) 12.50 years old]	Clinical setting	AVG The intervention with the Boccia-Wii^®^ game was conducted over a period of two and a half months, with one and a half hours of weekly training, spread over 3 days a week, with 30 min duration each day. Duration: 10 weeks; 30 sessions.	- Range of motion and muscle activity; -Global strength of the superior extremity; - Global strength of the lower extremity; - Fine motor skills and Coordination; -ADLs (The Index Barthel); -HRQoL (EQ-5D test); -Self-esteem (Rosenberg Scale).	Statistically significant improvements were found in the following measures: fine motor skills of the hand and hand coordination, level of health-related quality of life, active range of movement in the elbow flexion, in the wrist extension, in the radial deviation, and in the biceps muscle activity.	10
Sevick et al. (2016) (United States)	Case Study	4 children with CP [10.8 (SD) 3.6 years old]	Home setting	AVG - Research assistants trained the parents to conduct the in-home sessions during the first 3 weeks; - Child performing 5 min of supervised EU stretching to warm-up. -Played 4 different games while standing (each ∼ 10 min) -Rest breaks Duration: 12 weeks	-Game Scores; -Platform records; -Movement sensor; -Active Range of Motion; - Manual coordination subtest of the Bruininks–Oseretsky Test of Motor Proficiency (BOT-2); - Modified UE Functional Targeting Reach Test; -Intrinsic Motivation.	The study showed changes in terms of motor function for some but not all participants of the study. In terms of other indicators, both the scores in the games and the number of repetitions per participant increased throughout the intervention.	10
Sandlund et al. (2011) (Sweden)	Repeated measures	14 children with CP (age range 6–16)	Home setting	AVG Daily practice with the EyeToy for PlayStation2 in their homes. The recommendation was for each child to practice by playing at least for 20 min each day. Duration: 4 weeks	-Physical activity monitors; -Motor Performance; -Upper limb co-ordination; General motor function (1 Minute Walk Test); -Gaming Diary: - Time spent on playing every day was recorded; -Interviews with parents.	Motivation for practice and compliance with training were high. Overall, there was an increase in children’s physical activity during the gaming weeks, translating in an increase in energy expenditure. Time spent as physically active increased. The two additional motor tests showed non-significant progress.	10
Yagüe Sebastián et al. (2016) (Spain)	A longitudinal, prospective experimental pretest posttest design type with one group	8 children with CP (range 6-12 years old)	Clinical setting	AVG -15 individual sessions with the child of 30 min each;- Each game (in a total of three) is played 2 times per child (one with the therapist and another alone); -Create a custom profile; - Draw avatar to provide feedback.	-Pediatric Balance Scale; - Comparison of the distribution of loads in both lower extremities with the Wii Balance Board; - Score on an ad hoc scale of satisfaction of the physiotherapy sessions to the user; -Games records.	Improvements in the satisfaction with the therapy, balance and postural control scores, as well as a more homogeneous distribution of weight load between the two lower-limbs.	10
							
Yalon-Chamovitz and Weiss (2008) (Israel)	Quasi-experimental	33 participants with CP [28.1 (SD) 5.3 years old]	Rehabilitation setting	AVG Experimental group played VR activity 30 min, 2/3 times a week for a period of 12 weeks.	- GestureTek GX single camera-based video capture VR system (GX); - Participants feedback questionnaire; - Instructor structured observation; -Self-esteem questionnaire; -Interviews.	Participants self-reported high enjoyment with the VR games, and this was stable throughout the intervention. Moreover, participants self-report of success was high and perceived as similar across games.	13
Winkels et al. (2012) (Netherlands)	Explorative clinical trial, with a pre- and post-measurement	15 children with CP (*M* = 8.9 years old)	Clinical setting	AVG Children attended training with the Wii home video game console Sports twice a week at the RRC. Each session took 30 min in which they played both boxing and tennis for 15 min holding the controller in their most-affected arm. Duration: 6 weeks. Physical therapists trained the children.	- Melbourne assessment of unilateral upper limb function. - ABILHAND-Kids (functional scale); - User satisfaction questionnaire; - Health professional usability questionnaire; - Visual analogue scale (assess enjoyment)	The study obtained mixed results. Particularly, although no changes in the upper-limb function were observed after the intervention, except for more affected children who revealed some improvements, the activities of daily living were more easily performed as rated by the participants’ caregivers.	10
Kassee et al. (2017) (Canada)	Pilot study employed a pre-test, post-test experimental Design (with follow-up measures)	6 male children with CP (range = 1–12 years old)	Home setting	AVG Participants were instructed to play the Wii using their affected (spastic) hand for at least 40 min each day, 5 days a week for 6 weeks (30 days). Participants were then given a series of 6 exercises to do at home, at an intensity of 12 repetitions per exercises, for two sets (i.e., corresponding to 24 repetitions for each exercise), 5 days a week, for 6 weeks.	- Melbourne Assessment of Unilateral Upper Limb Function-2 (Melbourne-2); - The ABILHAND-Kids questionnaire; - Daily logbook (Compliance, parent feedback, and feasibility); -Parental questionnaire.	The results showed that participants in the Wii group had a higher compliance rate with the prescribed training than the participants in the resistance group.	12

#### Inclusion and Exclusion Criteria

In the previous phases, papers were included if they met the following inclusion criteria: the paper must (i) include any study in which the majority of the intervention sample had CP (i.e., intervention sample composed of more than 50%); (ii) include any form of GUS (e.g., interactive computer-based game software) for one or multiple players, for any platform, as long as it was in an intervention; (iii) include an assessment of the intervention’s outcomes (i.e., quantitative/qualitative); and (iv) published in a peer-reviewed journal. Papers were excluded when (i) they were not research articles, but rather, they were reviews, editorial or commentaries; (ii) they were not written in Portuguese, Spanish, or English languages; and finally, (iii) they were methodology focused or technical only.

### Data Analysis

Each reviewer (SL, PM, and AP) assessed the selected articles and extracted the relevant information regarding the game used with serious purposes in an intervention context as well as the assessment of their effectiveness. The goal was to learn about the role that GUS play in interventions involving patients with CP. **Table 1** summarizes this information.

### Quality of the Studies

A code scheme based on Connolly et al. (2012) was used to assess the quality of the articles included in the review. The evaluation of the 79 papers was made by two researchers (SL and PM) independently. Moreover, each paper was assessed in five dimensions using a three point Likert scale. The final score was obtained by summing all scores in each of the 5 dimensions (ranging between 5 and 15, lowest and highest score, respectively). Problematic coding and discrepancies were discussed and jointly resolved. The inter-rater reliability (r) for the total scores was 0.89, denoting a good agreement between researchers about the quality of the papers.

## Results

**FIGURE 1:**
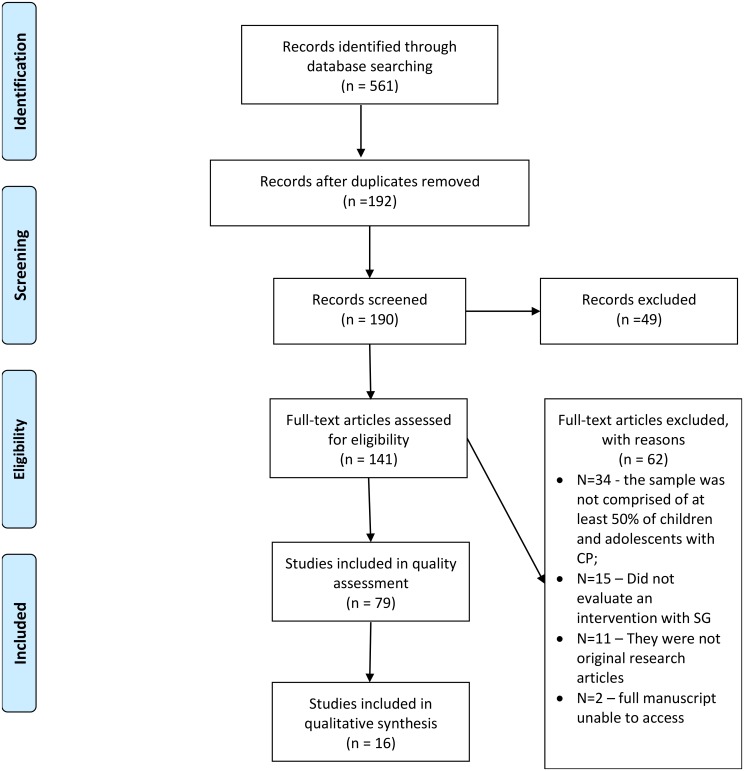
PRISMA Flow Diagram: Flow of information through the search and selection process during this systematic review.

The searches conducted in April of 2018 resulted in 561 papers total, of which 369 were duplicates, with 192 articles remaining (**Appendix A**). After the title selection, 190 studies remained and abstracts were screened for eligibility. At this stage, 49 articles were excluded by abstract screening. Therefore, 141 publications were retrieved for assessment against the inclusion and exclusion criteria and 60 did not comply with the criteria. Additionally, authors were unable to access the full manuscript of 2 publications (García-Vergara et al., 2013; Lin, 2014). **Figure 1** outlines these results by showing the flow of information throughout the different phases of this systematic review. This flow figure followed the PRISMA guidelines (Moher et al., 2009).

Seventy-nine papers were included for quality assessment. Each paper included was assessed in terms of its quality, as described in section “Quality of the Studies” (Connolly et al., 2012). After scoring each individual paper, authors established that only papers scoring 3 out of 4 on the fourth criterion (*Is the study purpose relevant for the review?*) could be included in the present review. To score 3 in this criterion, papers had to include an intervention, use GUS, and include a formal assessment of the motivation/ enjoyment/engagement in the intervention. After this selection, 30 papers remained and the mean score and mode were calculated (*M* = 9.67; *SD* = 1.73; Mode = 10), with scores ranging between 7 and 15 points. Following Connolly et al. (2012), papers were considered in this review using the mode as a cutoff point. Papers with scores 10 or above were included in this review because they are more likely to provide methodologically robust evidence of the impact of GUS in interventions involving patients with CP. Finally, of the 30 papers, 16 individual studies were included in the final review (see **Appendix B**).

### Organization of the Studies

**Table 1** summarizes the general characteristics of the sample of studies, including the reference, sample, purpose of the intervention, game used, measures, results, and conclusions. In sum, this review included 16 studies with a total of 203 participants with CP. The majority of the studies were conducted with children/adolescents (e.g., Howcroft et al., 2012a); and only three were conducted with adults (Yalon-Chamovitz and Weiss, 2008; Diez Alegre and Cano de la Cuerda, 2012; Jaume-i-Capo et al., 2014).

Studies sharing the main purpose of assessing the effectiveness of an intervention that incorporates games and/ or VR to promote a certain skill (e.g., improve balance) were included in this category (Yalon-Chamovitz and Weiss, 2008; Sandlund et al., 2011; Diez Alegre and Cano de la Cuerda, 2012; Howcroft et al., 2012a,b; Ramstrand and Lygnegård, 2012; Winkels et al., 2012; Burdea et al., 2013; Jaume-i-Capo et al., 2014; Preston et al., 2015; Sevick et al., 2016; Yagüe Sebastián et al., 2016; Kassee et al., 2017; Levac et al., 2017; Liu et al., 2017; MacIntosh et al., 2017). These papers were further assigned into two sub-categories related to the setting in which the intervention takes place. Thus, papers were classified as *home-based interventions* when the purpose was to evaluate the efficacy of autonomously playing a game, or a set of games, at home; and as *clinical-setting interventions* when the purpose was to evaluate the efficacy of playing a game, or a set of games, at a clinical setting.

#### Home-Based Interventions

##### Purpose and design of the studies

Of the 16 interventions included in the present study, seven took place at the patients’ home (Sandlund et al., 2011; Ramstrand and Lygnegård, 2012; Preston et al., 2015; Sevick et al., 2016; Kassee et al., 2017; Levac et al., 2017; Liu et al., 2017). These seven studies demanded patients’ to be self-governing and followed the prescribed “diet” of autonomous play, with little or without assistance and guidance of a therapist. Four studies aimed to improve the upper-limb/extremity function (two used COTS games, one used purposively developed games, and one study did not mention the nature of the games used). One study aimed to promote functional mobility (with COTS); the other study aimed to improve balance (with COTS); and the last study aimed to promote physical activity and motor performance (with COTS). Thus, five out of the seven studies used COTS games in their intervention.

Four of the seven home-based studies aimed to promote upper-limb/extremity function. For example, Kassee et al. (2017) goal was to examine the potential of Nintendo Wii Sports Resort Games’ intervention to improve upper-limb function compared with traditional resistance training. For both groups, the training lasted for 6 weeks, with a pre-post-follow-up design. Participants of the Wii group were asked to play with the spastic hand for at least 40 min per day, 5 days per week; whereas participants of the resistance training were asked to do a set of six exercises that trained the same functions as the games played by the Wii group, with a frequency and number of repetitions that are equivalent to the 40-min play. Parents supervised the training and had the task of recording information in a logbook regarding the time of day, the duration, and the games/exercises completed by the child. Regarding Liu et al. (2017) intervention their goal was twofold. First, to examine the feasibility, usability, and acceptability of an electromyography with accelerometers (EMG-ACC) game-based upper limb training system to be used at home in the future, which will be addressed in this section. Second, to assess the effect of using the system in a long-term game intervention training. Overall, to achieve their goals, authors measured an array of physiological indicators and self-reported game experience to assess if the tools selected can be used with a therapeutic purpose. The games were purposely designed and developed for this two-stage intervention. Stage 1 lasted for 1 month and participants had to play for 30 min, 2–3 times per week. Stage 2 lasted for 1.5 months and participants had to play for 30 min, 1 time per week. In the study by Preston et al. (2015), the goal was to examine the benefit of using a computer-assisted arm rehabilitation gaming technology on arm function. The study was a 6-week single-blind randomized controlled trial with a 12-week follow-up. The intervention included a control group, receiving treatment as usual, and an assistive device group, receiving treatment as usual plus the assistive games device at home. Participants in the gaming group were asked to play for 30-min a day. Parents were invited to partake in the intervention by assuming the role of encouraging their child to play and of verifying the gaming technology. Sevick et al. (2016) study aimed to examine the feasibility of using free internet videogames to promote upper extremity motor function and to assess the degree of motivation of the participants during the intervention. This intervention used the Kinect motion sensor together with the Flexible Action and Articulated Skeleton Toolkit software (FAAST). It lasted for 12 weeks (about 1-h training per week), comprising a total of 26 COTS games. Games could be played at children’s discretion based on personal interest, provided the target body areas remained constant. Moreover, parents received training by the researchers to conduct the home-based training sessions.

In the study by Levac et al. (2017), authors wanted to compare participants’ changes in Gross Motor function and functional mobility as a function of the intervention group they were assigned to. The two groups included a 6-week online monitored intervention using *Kinect Xbox 360* COTS active videogames, during which participants were expected to play for 30 min, 5 days a week. Additionally, one of the groups received an additional 1-week intensive training with a VR system in the clinical setting prior to the 6-week program. The intervention was supervised online by a physical therapist, on a website purposely built so that participants and their families could log information about actual game-play and performance, and could communicate with the therapist. Progression and adjustments to the games were performed by the physical therapist based on the information logged on the website. In the study by Ramstrand and Lygnegård (2012), the aim was to assess the efficacy of playing *Wii* Fit COTS games to improve balance. This intervention was unsupervised, as the only requirement was that participants played for 30 min, 5 times per week, for 5 weeks. Because the games used were COTS, no calibration or adaptation was deemed necessary. Lastly, Sandlund et al. (2011) tested the possibility of using COTS games with an interactive system based on video-capture technique as promoters of physical activity and motor performance. In this motion interactive system, participants respond to the stimulus of the game with body movements (e.g., jump, run on the spot, balance) and watch themselves in the screen. Participants played this COTS games in the *PlayStation 2* for 20 min each day, during 4 weeks.

##### Assessment

Considering the goals of the studies included in this category, the measures selected to assess the efficacy of the interventions were circumscribed to the skills trained in the interventions. Nevertheless, besides the quantitative measures of the target skill, the majority of the studies also included qualitative measures, such as interviews or diary logs, to further understand the impact of the intervention on the participants and their families.

To assess the efficacy of their intervention in promoting upper-limb function, Kassee et al. (2017) collected the following measures: Melbourne Assessment of Unilateral Upper Limb Function-2 (Melbourne-2), the ABILHAND-Kids questionnaire, and the average maximal grip strength. Complementary, other measures were collected, including: daily logbook, which evaluated compliance, motivation, and feasibility; and a parental questionnaire to assess motivation and feasibility of the intervention. Liu et al.’s (2017) assessment protocol was focused on assessing the usability of the rehabilitation system developed. Thus, self-reported measures assessing the comfort, understandability, ease, efficacy of feedback, interestingness, attention, and fatigue was completed by the participants. Still, the intervention used several measures to assess upper limb rehabilitation: the upper-extremity subsection of the Fugl-Meyer Assessment Scale, an upper-extremity ADL scale, and participants’ performance in each game. Regarding the Preston et al. (2015), the assessment included the following measures: the ABILHAND-kids and the Canadian Occupational Performance Measure. This data was complemented with information provided by the families in the family diaries where parents were asked to describe the rehabilitation process, which also included a questionnaire evaluating the following aspects: use of the gaming system, engagement with games, and participation in the study. The study by Sevick et al. (2016) used the following measures to assess upper extremity motor function: Active Range of Motion, the manual coordination subtest of the Bruininks–Oseretsky Test of Motor Proficiency (BOT-2), and the Modified UE Functional Targeting Reach Test. Additionally, Intrinsic Motivation during training was assessed two times per week with the interest/enjoyment subscale of the IMI. Moreover, all utterances emitted by the participants during game play were registered.

The Levac et al. (2017) intervention included an array of repeated-measures that were collected in every session. These measures included: the assessment of postural responses (Computer Assisted Rehabilitation Environment); the Six Minute Walk Test; Gross Motor Function Measure Challenge Module; and Participant perceptions of the AVG exercise program. Ramstrand and Lygnegård (2012) intervention used several measures to assess balance (e.g., modified sensory organization test), as well as diaries in which participants had to record their playing time. Finally, Sandlund et al. (2011) assessed the efficacy of playing games to promote physical activity and motor performance with an array of tasks and measures (e.g., SenseWear Pro3 Armband to measure energy expenditure), a gaming diary completed by the participants, and interviews with the parents to understand the family’s experience during the intervention.

##### Main results and conclusion

There were mixed results related to the home-based interventions in terms of effectively training the skills targeted and with some therapeutic benefits. Still, the majority of the studies reported high levels of compliance, motivation, and overall engagement with the game-based intervention.

Results of the study by Kassee et al. (2017) showed that participants in the Wii group had a higher compliance rate with the prescribed training than participants in the resistance group. Moreover, parents of children allocated in the Wii group were more positive, more motivated, and considered the intervention more feasible than the resistance group. Despite that results regarding the functional measures were not conclusive, there were still some improvements among the participants of the Wii group. In the study by Liu et al. (2017), the majority of the participants reported feeling motivated to play the games and being fatigued at the end of practicing. Additionally, the guardians of the participants reported that the majority of the participants were able to focus for longer periods of time on the game-based training compared with the conventional training. All participants improved their gaming performance and motor control. No changes were observed in relation to muscle strength. The study by Preston et al. (2015) failed to observe improvements in the arm function. Authors attribute this lack of improvement to the low compliance with the prescribed training regimen, particularly regarding the frequency and duration of game play. Despite children’s interest in the games, authors speculate that four distinct games may not suffice to maintain interest levels high. Thus, game-based training should be regarded as a complement of traditional rehabilitation sessions, particularly with the possibility of including competitive and collaborative play. Sevick et al. (2016) study showed changes regarding motor function for some but not all participants. Moreover, both the scores in the games and the number of repetitions per participant increased throughout the intervention; participants’ motivation levels were high and sustained throughout the intervention. Authors concluded that these sort of games can be used in the therapy context to foster patients’ motivation and engagement in therapy.

The intervention by Levac et al. (2017) showed no differences between groups regarding functional mobility after the 6-week training with AVG. Still, the group that did not receive prior-intervention intensive training with a VR system played more time each day than the other group. This may have contributed to enhance the Gross Motor function observed in this group after the 6-week intervention. Additionally, participants’ perceptions of the AVG exercise program (enjoyment, fatigue, ease, difficulty, and boredom) remained fairly stable across weeks, suggesting that the individualized games’ adjustments performed by the therapist maintained the degree of challenge. Ramstrand and Lygnegård (2012) showed that playing the COTS video games for *Wii* had no effect on balance. Nevertheless, the authors reflected on the fact that it is difficult to ensure participants’ compliance with the intervention protocol in home-based interventions. The last intervention, by Sandlund et al. (2011), promoted an increase in physical activity among the participants. Moreover, inspection of their gaming diaries indicated that they felt motivated to practice. Still, authors observed that the interest in playing the games diminishes over time and suggest that game-based interventions may be more suitable for short-term intensive interventions.

Overall, authors suggested that these tools could complement usual therapy to help achieve and maintain the therapeutic goals. Moreover, interviews with the participants and their families, as well as family diary logs, indicated that games were motivating enough to keep them engaged with the prescribed protocol. Still, some authors alerted to the decreased interest in continuing to play the games after a few weeks into the intervention. This finding highlights the need to keep these sort of interventions short and intensive, or to have a large and diversified set of games to maintain interest. Finally, in these home-based studies, families played a key role in controlling the therapeutic process. For example, families could schedule when and for how long the participants could play. Families also helped motivate the patients to persist in the task. The emergence of inter-family reflections regarding the importance of including games in the intervention program was an outcome of family involvement.

#### Clinical-Setting Interventions

##### Purpose and Design of the Studies

Of the 16 interventions included in the present study, nine took place in a clinical-setting (Yalon-Chamovitz and Weiss, 2008; Diez Alegre and Cano de la Cuerda, 2012; Howcroft et al., 2012a,b; Winkels et al., 2012; Burdea et al., 2013; Jaume-i-Capo et al., 2014; Yagüe Sebastián et al., 2016; MacIntosh et al., 2017). Regarding their purpose, two studies aimed to improve upper extremity function and both used COTS games for *Wii*. Two other studies aimed to improve balance; one developed SG to attain its aim and the other study used COTS games for *Wii*. One study aimed to promote lower extremity function with games specifically developed for its VR-system. Another study aimed to evaluate the efficacy of three algorithms designed to minimize differences in game success, time above 40% of heart rate reserve, and enjoyment, between participants with different levels in the Gross Motor Function. The first aimed to evaluate the possibility of using VR to enable participation in leisure activities. Finally, the last two studies aimed to examine the potential of active video games to act as promoters of physical activity.

Two out of the 9 studies aimed to improve upper extremity function. Specifically, Diez Alegre and Cano de la Cuerda (2012) purpose was to assess the efficacy of playing Boccia *Wii* to improve motor function of the upper extremity, including fine motor skills and hand coordination, with adults with CP. Their intervention took place three times per week, for a total of 30 sessions (30 min each). This intervention did not replace usual rehabilitation therapies. Regarding Winkels et al. (2012) study, the aim was to assess the possibility of using Nintendo Wii sports to train upper extremity function, namely the most affected arm, with the aid of a physical therapist. The intervention took place in the rehabilitation center for 30 min, two times per week, for 6 weeks.

Moreover, two other studies aimed to improve participants’ balance. Jaume-i-Capo et al. (2014) aim was to use SG to promote balance rehabilitation with adults with CP. Following the therapists’ guidelines, authors developed SG that could be controlled by the participants’ body movement, which were detected by *Microsoft Kinect*. The intervention lasted for 24 weeks, and participants had at least a 20-min session per week. This was the only physical therapy that these patients were attending to, as they were selected exactly because of their lack of adherence to conventional therapies. The study by Yagüe Sebastián et al. (2016) aimed to evaluate the possibility of including videogames in the physiotherapy sessions to improve balance, postural control, and a symmetric distribution of weight load between the lower-limbs. The Nintendo Wii-fit with the balance board was used to this aim. Each participant played for 30 min per session, for 15 sessions along a 3-months’ time-frame. In each session, each of the three games was played twice, one with the help of the physiotherapist and the other autonomously.

Burdea et al. (2013) intervention aimed to promote lower extremity motor function, including ankle strength and motor control, as well as the quality of life by using the Rutgers Ankle Robot. The Ankle Robot allowed participants to control the games. Training sessions were 40-min long, three times per week, for 12 weeks. The therapist was present for the set up and for the progression of each game. The study by MacIntosh et al. (2017) aimed to evaluate the efficacy of three algorithms designed to minimize differences in game success, time above 40% of heart rate reserve, and enjoyment between participants with different levels in the Gross Motor Function. The intervention lasted for six sessions and used three custom-built videogames that were played for 10-min each. Finally, Yalon-Chamovitz and Weiss (2008) purpose was to evaluate the possibility of using VR to enable participation in leisure activities with adults with CP. The training sessions occurred in a day-care facility, using the GestureTek GX (a single camera-based video capture VR system), for 30-min sessions, two to three times per week, for 12 weeks.

Both studies by Howcroft et al. (2012a,b) share the main purpose of examining the potential of active video games to act as promoters of physical activity, operationalized as energy expenditure, in both solo and multiplayer conditions. That is, the goal of these studies is not to assess the effectiveness of an intervention that incorporates games in its design, but to assess whether a particular tool (i.e., active videogames) would promote physical activity. In the Howcroft et al. (2012b) research the child played each AVGs (Wii bowling, Wii tennis, Wii boxing, DDR Disney Dance Grooves) in a randomized order for 8 min with a rest period of 5 min between each game during one session. Along the same line, Howcroft et al. (2012a) reported that the child played an AVG (“Wii Boxing”) twice, once against the computer and a second time against a member of the research team. The game was played for 8 min in both scenarios with a 5-min rest in between.

##### Assessment

Considering the goals of the studies included in this category, the measures selected to assess the efficacy of the interventions were circumscribed to the skills trained in the interventions.

Regarding the studies aimed to promote upper extremity function, several distinct measures were taken. Diez Alegre and Cano de la Cuerda (2012) included the muscle activity (e.g., electromyography), fine motor skills and coordination (Nine-hole Peg Test), as well as the self-esteem of the participants in their assessment protocol. Separately, Winkels et al. (2012) included measures to assess the upper-limb function (Melbourne assessment of unilateral upper limb function and ABILHAND-Kids) as well as instruments to measure satisfaction with the training (children and health professional), enjoyment (children), and its usability (health professional).

Regarding the studies aimed to promote balance, several distinct measures were taken. The study by Jaume-i-Capo et al. (2014) included the Berg Balance Scale, the Functional Reach Test, and the Balance Tinetti Test. Additionally, a member of the research team a member filled out a daily case report form in which information was recorded about participants’ volition, among other features. Whereas the Yagüe Sebastián et al. (2016) study included the Pediatric Balance Scale, Wii Balance Board provided data of on weight load distribution, and a motivation and satisfaction scale.

To assess the lower extremity motor function after an intervention with the Rutgers Ankle Robot, Burdea et al. (2013) used a diverse set of measures, including Gross Motor Function Measure, ankle strength and maximum dorsiflexion at initial contact during gait, gait speed, and quality of life. To assess the effectiveness of the algorithms in minimizing differences in game success, time above 40% heart rate reserved, and enjoyment, MacIntosh et al. (2017) selected the following measures: game success, playtime percent above the 40% heart rate reserve, and Intrinsic Motivation Inventory Questionnaire. Finally, to assess the possibility of participation in leisure activities, Yalon-Chamovitz and Weiss (2008) included the following measures: information about the VR system (enjoyment, presence and control, feelings of discomfort) collected at the end of each session; structured observations by the instructor; and a pre-post evaluation of participants’ self-esteem.

Considering the aim that underlies the studies by Howcroft et al. (2012a,b) and their laboratorial setting, the assessment conducted was complex and involved the use of several physiological measures. Because of this, calibration and adaptation of the instruments of measurement to each participant were necessary. Additionally, in each study, the physiological measures were carefully selected to capture the effect under scrutiny. Thus, the emphasis of the assessment was not on the outcomes of the game played, but on the effectiveness of the game in eliciting the desired activity. In both studies, the assessment protocol implied the use of anthropometric, energy expenditure, motion capture, and muscle activity measures.

##### Main results and conclusion

Most studies included in the *Clinical-setting Interventions* reported gains regarding the target skill trained.

The results by Diez Alegre and Cano de la Cuerda (2012) were mixed. After the intervention, there were clinical improvements in the coordination and fine motor skills of the upper extremities. However, there were no differences in terms of independence in performing the activities of daily living. Authors recommend the use of more sensitive measures to capture small improvements in the performance of the activities of daily living among this population. Similarly, Winkels et al. (2012) reported mixed results. Particularly, no changes in the upper-limb function were observed after the intervention, except for the case of children with lower functionality who revealed some improvements; however, participants’ caregivers reported that the activities of daily living were more easily performed by participants. Moreover, enjoyment and perceived effort were higher in Wii play condition when compared with the conventional therapy. Authors highlighted the potential of these instruments to be used at home as a way to train balance, and arm and trunk movements.

Jaume-i-Capo et al. (2014) paper reported that there was an improvement in participants’ balance in all the functional measures considered. Additionally, participants were able to understand that their actual movements had an effect in the virtual world. Authors concluded that these tools (e.g., SG) could be used successfully in the clinical setting. Similarly, Yagüe Sebastián et al. (2016) data revealed improvements in the balance and postural control scores, as well as a more homogeneous distribution of weight load between the two lower-limbs. Finally, there was an increased satisfaction with the physiotherapy sessions, with children preferring the sessions including videogames. Authors concluded that the use of Wii could complement the rehabilitation process.

Mixed results were found by Burdea et al. (2013). Different participants obtained distinct combinations of gains in the several measures used. Overall, Gross Motor Function Measure, gait function, and quality of life improved with the intervention. Although games were engaging, as evidenced by the high number of repetitions per exercise, authors stressed the need to diversify the games used in the rehabilitation context to keep patients motivated and engaged with the therapy. Authors suggested these sort of tools as possible complements to conventional rehabilitation that can be implemented at home. MacIntosh et al. (2017) also obtained mixed results regarding the three algorithms tested. None singled-out the other in minimizing differences in game success, time above 40% heart rate reserve, and enjoyment as a function of participants’ Gross Motor Function level. Still, authors underlined the benefits of using such methodologies of balancing the game parameters (through the algorithms tested) based on individual abilities. Finally, Yalon-Chamovitz and Weiss (2008) participants self-reported high enjoyment with the VR games, and this was stable throughout the intervention. Moreover, participants self-report of success was high and perceived as similar across games. These measures were always higher than the instructors’ perception of the participants’ enjoyment and success. Howcroft et al. (2012a,b) showed that active video games do elicit muscle activation and energy expenditure, and thus can be regarded as promoters of physical activity. These studies allowed authors to conclude that active video games can be incorporated into the daily routine of individuals with CP as a complement to the therapies, as the level of activity reached while playing the games was light to moderate physical activity. Considering that the games tested are COTS, individuals will benefit from an independent and autonomous play of these games without the need to calibrate or adapt them, or the presence of a therapist. Additionally, users enjoyed playing the games, which is an added benefit of these tools. Specifically, participants expressed their preference for playing in the multiplayer condition compared with the solo condition. Overall, Howcroft et al. (2012a,b) suggest that these tools could be alternatives or complements to usual therapy. Particularly, the studies emphasized the engaging, motivating, and fun aspects of interacting with these virtual tools (i.e., active video games), all critical aspects to guarantee adherence to the therapy and rehabilitation and assure that the practice of the exercises prescribed continues at home. Still, authors advise caution when using the multiplayer function and suggest the solo play in the therapy context. Although participants enjoyed the multiplayer condition the most, authors observed that under this condition the use of the hemiplegic arm decreased and the use of the dominant arm increased when compared with the solo condition; thus suggesting this may not be the most appropriate scenario to reach the therapeutic goals.

Overall, the authors argue that research is still missing in order to assure that these game-based interventions could replace conventional therapies. Instead, the authors suggested that these tools could be a supplement of regular therapies, mainly due to their engaging nature.

### Overview of the Studies

In the majority of the studies, the interventions consisted solely of playing games; that is, games were not an adjunct to the intervention, they constituted the core of the intervention. In the majority of the studies, there was a therapist aiding the participant; only home-based interventions attempted individual, autonomous training.

Regarding the games’ features, overall not much effort was devoted to describing the characteristics of the games. That is authors specified whether games were COTS, or if they were games purposely designed for the intervention. Still, information on whether participants would keep a record of their performance or whether the game would continue on from the previous session was not provided. Nevertheless, considering that several interventions employed COTS games, and some took place at the participants’ home, we cannot rule out the possibility that at least in these situations participants could have had access to this sort of information if they wished to do so.

Furthermore, the purpose of the interventions included in this study was not focused on testing the efficacy of a particular game in training a particular skill, but rather on testing the efficacy of games in promoting a certain activity or skill and the possibility of being part of a rehabilitation scheme. Thus, the emphasis was not on the game itself, but rather on the fact that the intervention used games, contrasting with conventional intervention. Additionally, games and games’ outcomes were not used as measures of the quality of the movements, they were employed as a mean to practice the movement or skill. To measure the quality of the movements, and the impact of playing the games on the improvement of the movement or skill, each study selected an array of standardized measures.

## Discussion

This systematic review aimed to understand the role of GUS in interventions with patients with CP. The 16 studies reported interventions conducted either at home or in a clinical setting. Regarding the home-based interventions (Sandlund et al., 2011; Ramstrand and Lygnegård, 2012; Preston et al., 2015; Sevick et al., 2016; Kassee et al., 2017; Levac et al., 2017; Liu et al., 2017), the main purpose was to evaluate the efficacy of playing a game or a set of games autonomously, in order to promote skills at home with little or without supervision of a therapist. Four of the seven studies in this category aimed to train the upper-limb/extremity function, and the other three aimed to promote functional mobility, balance, and physical activity. There were mixed results related to the efficacy of the intervention in improving the targeted skill. Still, the majority reported some degree of therapeutic gains and high levels of motivation and engagement with the intervention. Additionally, home-based intervention promoted inter-family member reflections and involvement with the participants’ therapy. The findings highlighted that the family members or caregivers played an important role in the intervention’s success (Sandlund et al., 2011). Moreover, due to the portability of COTS games, interventions can be conducted at home while facilitating family organization and reducing costs for family members and caregivers. Family involvement in the therapeutic process highlights some major advantages of the use of games in interventions. Family members and caregivers can help control and manage the intervention at home by providing support to the patients as they train specific competences. Finally, the clinical-setting interventions’ (Yalon-Chamovitz and Weiss, 2008; Diez Alegre and Cano de la Cuerda, 2012; Winkels et al., 2012; Burdea et al., 2013; Jaume-i-Capo et al., 2014; Yagüe Sebastián et al., 2016; MacIntosh et al., 2017) purpose was to evaluate the efficacy of playing a game, or a set of games, in a clinical setting, usually under the supervision of a therapist. Two studies aimed to improve the upper extremity function; two other studies aimed to improve balance; one study aimed to promote lower extremity function; another study aimed to evaluate the efficacy of three algorithms designed to minimize differences in game success, time above 40% of heart rate reserve, and enjoyment between participants with different levels in the Gross Motor Function; and finally, the last study aimed to evaluate the possibility of using VR to enable participation in leisure activities. All studies reported some therapeutic gains with the inclusion of games in their intervention scheme. However, results regarding the evaluation of improvement of the target skills were mixed within studies and were dependent on the specific measure being used. For example, standardized measures may not capture the changes reported and observed by therapists and family in the daily lives of the participants.

Overall, the present systematic review allows authors to conclude that GUS can be effectively incorporated into conventional interventions, with clear therapeutic gains. These findings are consistent with data from systematic reviews assessing the efficacy of games in promoting skills, such as perceptual, cognitive, behavioral, affective, motivational (Connolly et al., 2012), and motor skills development (Page et al., 2017), in both typically developing youngsters (Connolly et al., 2012) and non-typically developing youngsters (Page et al., 2017). Regarding some aspects of the intervention, each had its own features, still, concerning the intervention length, the findings showed that the majority of studies performed more than five sessions (e.g., Sandlund et al., 2011), each lasting 30 min (e.g., Diez Alegre and Cano de la Cuerda, 2012; Burdea et al., 2013). Additionally, the main benefit of interventions that integrate GUS is their ability to increase the participants’ enjoyment in the intervention as well as their motivation to continue with it. This is particularly important because, as Bonnechère et al. (2014b) state, patients with CP, particularly children/adolescents, must strictly adhere to a rehabilitation scheme. Nevertheless, as the majority of the authors emphasized (e.g., Burdea et al., 2013), these game-based interventions should be used with caution and as a complement to the conventional therapies, not as a substitute. These therapeutic gains are related with the game-elements that make them engaging, motivating, and fun (e.g., Bonnechère et al., 2014a,b; Chiu et al., 2014; Li and Lwin, 2016). These characteristics of games add key-elements to conventional therapy and sustain patients’ interest in the therapy for longer periods of time. Still, some authors suggested that game-based interventions should be used in short-intensive interventions or that a large game-set should be available to help maintain interest in playing the games.

Regarding the types of games included, both COTS and SG were effective in training the target skills and motor functions. The possible advantage of using COTS is that they are cheap, accessible, there is no need for calibration, and can thus be used autonomously at home by the patients. Games that are developed to train a specific function and/ or that require the use of equipment tend to need calibration and the presence of a therapist in order to be used. This aspect can be a limitation if the goal is to have patients train a movement at home. However, it does have the added benefit of being tailored to train a particular skill or movement.

Finally, present results highlight that, despite their differences in nature (entertainment *vs.* non-entertainment), COTS and SG can be integrated into the same concept when the emphasis shifts from the nature of the game to the purpose of the use. Therefore, we suggest a new concept – GUS – to focus on the purpose of the use of the games, rather than on the nature of the game.

### Implications for Practice

The results of the present review shed some light on possible solutions to promote patients’ engagement in the therapy and potentiate therapeutic gains. Specifically, practitioners often struggle to get their patients to complete the assigned homework tasks, as patients display low motivation to engage in prescribed exercises. Thus, the inclusion of GUS as a complement to conventional therapy, in the format of homework assignments to be carried out outside the therapy at home, may increase the patients’ compliance with the exercises prescribed. Additionally, the selection of game(s) to be played at home is expected to match the patients’ needs. Moreover, the practitioner must monitor the compliance with the prescribed tasks at a regular basis, which will also inform of the difficulties found and progression in the games. Also, it is important to have a diverse set of games available in order to prevent decrease in motivation over time. Lastly, the present review underlined the major contribution that families may give to the success of the rehabilitation, stressing the need to always involve the family in the intervention schemes. For example, play-time at home may be shared with parents, siblings, and other members of the family; these family play moments are likely to contribute to the rehabilitation of their family member without being perceived as a burden.

### Limitations

The present review acknowledges that it has limitations. One of the limitations is transversal to all systematic reviews, which is that the outcomes of the search are restricted by the search terms and refinements used (i.e., journals included and time-period of publications). Although the systematic review may not accurately reflect all extant literature relevant to this study, it still provides a picture of the current research outcomes and the impact of embedding games into intervention schemes. Additionally, review and opinion studies were not included in the review as the goal was to systematize results arising from evidence-based studies. Another limitation is that despite the quality criteria of PRISMA and the authors’ abidance to the Cochrane guidelines to guarantee some methodological rigor, the authors cannot completely control the publication bias and therefore cannot guarantee full access to the data in the realm of this systematic review. A further limitation is that studies had to be written in Portuguese, Spanish, and English.

### Future Studies

Considering that the impairments of CP extend beyond the motor domain to deficits at the cognitive, executive function, and self-regulation level, current authors suggest that this could be a future avenue of investigation for researchers and practitioners. Considering the potential of games in domains other than motor rehabilitation (i.e., learning, teaching, training, and informing purposes) (Susi et al., 2007; Moyà Alcover et al., 2011) and its abilities to improve attention, memory, and performance in general executive functions (Boot et al., 2008; Colzato et al., 2012), future research could consider extending the use of games to exercise cognitive competences. For example, extant research has reported that children with CP are at a high risk of showing learning difficulties throughout school (Jenks et al., 2009, 2012). Hence, tackling these difficulties with tailored interventions using games may help to improve the efficacy of the interventions aimed at fostering the academic achievement of children with CP (e.g., training self-regulated learning strategies, see Núñez et al., 2013; Rosário et al., 2013, 2016).

Future studies could also consider testing specific combinations of game elements to analyze which are the most critical to intervention efficacy. For instance, including gamification [i.e., the use of game elements in non-game contexts (see Deterding et al., 2011)] in an intervention could elicit positive gains similar to those accomplished by including GUS. Another avenue for future research could be the comparison of the efficacy of an intervention conducted at home against the same intervention conducted at a rehabilitation center.

## Conclusion

This systematic review aimed to examine the role of GUS in interventions involving individuals with CP while specifically examining the purposes for including games in the intervention scheme, which games were used, how the efficacy of the intervention was assessed, and the main results and conclusions that were reached. The findings indicated that the main purposes for including games in the interventions were for motor rehabilitation. All studies, except for one, reported positive gains with the inclusion of games in their intervention scheme. Additionally, authors suggest that games could be included as complements to conventional therapy, especially due to their engaging and fun nature. The present review itself poses as a benefit to extant research as a possibility of being a guide to practice. For example, a therapist wanting to include a game into a session may consult **Table 1** to select the game, measure, and the protocol most appropriate to their goal.

## Author Contributions

SL was responsible for the literature search and data extraction. SL, PM, and AP were responsible for the blind literature search and the data extraction. CM and JM oversaw data interpretation and technical guidance. PR and EC made an important intellectual contribution to the research design and manuscript revision.

## Conflict of Interest Statement

The authors declare that the research was conducted in the absence of any commercial or financial relationships that could be construed as a potential conflict of interest.
