# Supplementary material for: Games Used With Serious Purposes: A Systematic Review of Interventions in Patients With Cerebral Palsy
Source: Front Psychol. 2018 Sep 19;9:1712. doi: 10.3389/fpsyg.2018.01712 (PMC6156132; doi:10.3389/fpsyg.2018.01712)
Supplement: Supplementary file 1 [file Data_Sheet_1.docx]

Supplementary Material

Games Used with Serious Purposes: A Systematic Review of Interventions in Patients with Cerebral Palsy

Sílvia Lopes^1^, Paula Magalhães^1^, Armanda Pereira^1^, Juliana Martins^1^, Carla Magalhães^2^, Elisa Chaleta, and Pedro Rosário^1*^

*** Correspondence:** prosario@psi.uminho.pt

# Supplementary Tables

**Appendix A –** Full search strategy and results

| **Database** | **Number of papers identified in search** | **Number of papers meeting inclusion criteria** | **Number of papers meeting after quality assessment** |
| --- | --- | --- | --- |
| **IEEE** | **33** | **7** | **1** |
| **PsyInfo** | **25** | **11** | **2** |
| **PubMeb** | **83** | **34** | **10** |
| **Scopus** | **169** | **18** | **3** |
| **Web of Science** | **251** | **9** | **0** |
| **Total** | **561** | **79** | **16** |
