# Supplementary material for: Games Used With Serious Purposes: A Systematic Review of Interventions in Patients With Cerebral Palsy
Source: Front Psychol. 2018 Sep 19;9:1712. doi: 10.3389/fpsyg.2018.01712 (PMC6156132; doi:10.3389/fpsyg.2018.01712)
Supplement: Supplementary file 2 [file Data_Sheet_2.docx]

Supplementary Material

Games Used with Serious Purposes: A Systematic Review of Interventions in Patients with Cerebral Palsy

Sílvia Lopes^1^, Paula Magalhães^1^, Armanda Pereira^1^, Juliana Martins^1^, Carla Magalhães^2^, Elisa Chaleta, and Pedro Rosário^1*^

*** Correspondence:** prosario@psi.uminho.pt

# Supplementary Tables

**Appendix B**. Summary of Papers included for quality assessment.

| Alegre, M.I. Diez, and R. Cano de la Cuerda. “Empleo de un video juego como herramienta terapéutica en adultos con parálisis cerebral tipo tetraparesia espástica. estudio piloto.” Fisioterapia 34, no. 1 (January 2012): 23–30. doi:10.1016/j.ft.2011.09.001.* |
| --- |
| Atasavun Uysal, Songül, and Gül Baltaci. “Effects of Nintendo Wii™ Training on Occupational Performance, Balance, and Daily Living Activities in Children with Spastic Hemiplegic Cerebral Palsy: A Single-Blind and Randomized Trial.” Games for Health Journal 5, no. 5 (October 2016): 311–317. doi:10.1089/g4h.2015.0102. |
| Barton, Gabor J., Malcolm B. Hawken, Gill Holmes, and Michael H. Schwartz. “A Gait Index May Underestimate Changes of Gait: a Comparison of the Movement Deviation Profile and the Gait Deviation Index.” Computer Methods in Biomechanics and Biomedical Engineering 18, no. 1 (March 22, 2013): 57–63. doi:10.1080/10255842.2013.776549. |
| Bhakta, B, A Weightman, N Preston, M Levesley, R Holt, M Mon-Williams, M Clarke, and AJ Cozens. “Home Based Computer-Assisted Upper Limb Exercise for Young Children with Cerebral Palsy: A Feasibility Study Investigating Impact on Motor Control and Functional Outcome.” Journal of Rehabilitation Medicine 43, no. 4 (2011): 359–363. doi:10.2340/16501977-0679. |
| Bingham, Peter M., and Barbara Calhoun. “Digital Posturography Games Correlate with Gross Motor Function in Children with Cerebral Palsy.” Games for Health Journal 4, no. 2 (April 2015): 145–148. doi:10.1089/g4h.2014.0096. |
| Bonnechère, Bruno, Lubos Omelina, Bart Jansen, and Serge Van Sint Jan. “Balance Improvement after Physical Therapy Training Using Specially Developed Serious Games for Cerebral Palsy Children: Preliminary Results.” Disability and Rehabilitation 39, no. 4 (August 3, 2015): 403–406. doi:10.3109/09638288.2015.1073373. |
| Bonnechère, Bruno, Lubos Omelina, Bart Jansen, Marcel Rooze, and Serge Van Sint Jan. “Balance Training Using Specially Developed Serious Games for Cerebral Palsy Children, a Feasibility Study.” Proceedings of the 8th International Conference on Pervasive Computing Technologies for Healthcare (2014). doi:10.4108/icst.pervasivehealth.2014.255332. |
| Bulea, Thomas C., Zachary F. Lerner, Andrew J. Gravunder, and Diane L. Damiano. “Exergaming with a Pediatric Exoskeleton: Facilitating Rehabilitation and Research in Children with Cerebral Palsy.” 2017 International Conference on Rehabilitation Robotics (ICORR) (July 2017). doi:10.1109/icorr.2017.8009394. |
| Burdea, G. C., A. Jain, B. Rabin, R. Pellosie, and M. Golomb. “Long-Term Hand Tele-Rehabilitation on the Playstation 3: Benefits and Challenges.” 2011 Annual International Conference of the IEEE Engineering in Medicine and Biology Society (August 2011). doi:10.1109/iembs.2011.6090522. |
| Burdea, Grigore C., Daniel Cioi, Angad Kale, William E. Janes, Sandy A. Ross, and Jack R. Engsberg. “Robotics and Gaming to Improve Ankle Strength, Motor Control, and Function in Children With Cerebral Palsy—A Case Study Series.” IEEE Transactions on Neural Systems and Rehabilitation Engineering 21, no. 2 (March 2013): 165–173. doi:10.1109/tnsre.2012.2206055.* |
| Camara Machado, Fabiana Rita, Priscilla Pereira Antunes, Jandara De Moura Souza, Antônio Cardoso Dos Santos, Daniela Centenaro Levandowski, and Alcyr Alves De Oliveira. “Motor Improvement Using Motion Sensing Game Devices for Cerebral Palsy Rehabilitation.” Journal of Motor Behavior 49, no. 3 (September 3, 2016): 273–280. doi:10.1080/00222895.2016.1191422. |
| Chang, Yao-Jen, Wen-Ying Han, and Yu-Chi Tsai. “A Kinect-Based Upper Limb Rehabilitation System to Assist People with Cerebral Palsy.” Research in Developmental Disabilities 34, no. 11 (November 2013): 3654–3659. doi:10.1016/j.ridd.2013.08.021. |
| Chen, Kai, Yi-Ning Wu, Yupeng Ren, Lin Liu, Deborah Gaebler-Spira, Kelly Tankard, Julia Lee, Weiqun Song, Maobin Wang, and Li-Qun Zhang. “Home-Based Versus Laboratory-Based Robotic Ankle Training for Children With Cerebral Palsy: A Pilot Randomized Comparative Trial.” Archives of Physical Medicine and Rehabilitation 97, no. 8 (August 2016): 1237–1243. doi:10.1016/j.apmr.2016.01.029. |
| Chien-yu Lin, and Shu-hua Chen. “Effectiveness of Interactive Teaching Materials on Special Education and Assistive Technology.” 2014 9th International Conference on Computer Science & Education (August 2014). doi:10.1109/iccse.2014.6926479. |
| Chiu, Hsiu-Ching, Louise Ada, and Hsin-Min Lee. “Upper Limb Training Using Wii Sports Resort™ for Children with Hemiplegic Cerebral Palsy: a Randomized, Single-Blind Trial.” Clinical Rehabilitation 28, no. 10 (May 21, 2014): 1015–1024. doi:10.1177/0269215514533709. |
| Chung, Peter J., Douglas L. Vanderbilt, Sheree M. Schrager, Eugene Nguyen, and Eileen Fowler. “Active Videogaming for Individuals with Severe Movement Disorders: Results from a Community Study.” Games for Health Journal 4, no. 3 (June 2015): 190–194. doi:10.1089/g4h.2014.0091. |
| Curtis, Derek John, Jesper Bencke, and Bente Mygind. “The Effect of Training in an Interactive Dynamic Stander on Ankle Dorsiflexion and Gross Motor Function in Children with Cerebral Palsy.” Developmental Neurorehabilitation 17, no. 6 (May 27, 2014): 393–397. doi:10.3109/17518423.2013.844738. |
| Deutsch, J. E, M. Borbely, J. Filler, K. Huhn, and P. Guarrera-Bowlby. “Use of a Low-Cost, Commercially Available Gaming Console (Wii) for Rehabilitation of an Adolescent With Cerebral Palsy.” Physical Therapy 88, no. 10 (August 8, 2008): 1196–1207. doi:10.2522/ptj.20080062. |
| Do, Ji-Hye, Eun-Young Yoo, Min-Ye Jung, and Hae Yean Park. “The Effects of Virtual Reality-Based Bilateral Arm Training on Hemiplegic Children’s Upper Limb Motor Skills.” NeuroRehabilitation 38, no. 2 (February 18, 2016): 115–127. doi:10.3233/nre-161302. |
| Gallagher, Justin, Nick Preston, Raymond Holt, Mark Mon-Williams, Martin Levesley, and Andrew Weightman. “Assessment of Upper Limb Movement with an Autonomous Robotic Device in a School Environment for Children with Cerebral Palsy.” 2015 IEEE International Conference on Rehabilitation Robotics (ICORR) (August 2015). doi:10.1109/icorr.2015.7281295. |
| Gatica-Rojas, V., Méndez-Rebolledo, G., Guzman-Muñoz, E., Soto-Poblete, A., Cartes-Velásquez, R., Elgueta-Cancino, E., & Cofré, L. L. “Does Nintendo Wii Balance Board improve standing balance? A randomized controlled trial in children with cerebral palsy”. 2017. European journal of physical and rehabilitation medicine, 53(4): 535-544. doi: 10.23736/S1973-9087.16.04447-6 |
| Gatica-Rojas, Valeska, Ricardo Cartes-Velásquez, Guillermo Méndez-Rebolledo, Eduardo Guzman-Muñoz, and L. Eduardo Cofré Lizama. “Effects of a Nintendo Wii Exercise Program on Spasticity and Static Standing Balance in Spastic Cerebral Palsy.” Developmental Neurorehabilitation 20, no. 6 (August 18, 2016): 388–391. doi:10.1080/17518423.2016.1211770. |
| Gerber, Corinna N., Bettina Kunz, and Hubertus J. A. van Hedel. “Preparing a Neuropediatric Upper Limb Exergame Rehabilitation System for Home-Use: a Feasibility Study.” Journal of NeuroEngineering and Rehabilitation 13, no. 1 (March 23, 2016). doi:10.1186/s12984-016-0141-x. |
| Golomb, Meredith R., Brenna C. McDonald, Stuart J. Warden, Janell Yonkman, Andrew J. Saykin, Bridget Shirley, Meghan Huber, et al. “In-Home Virtual Reality Videogame Telerehabilitation in Adolescents With Hemiplegic Cerebral Palsy.” Archives of Physical Medicine and Rehabilitation 91, no. 1 (January 2010): 1–8.e1. doi:10.1016/j.apmr.2009.08.153. |
| Golomb, Meredith R., Stuart J. Warden, Elaine Fess, Bryan Rabin, Janell Yonkman, Bridget Shirley, and Grigore C. Burdea. “Maintained Hand Function and Forearm Bone Health 14 Months After an In-Home Virtual-Reality Videogame Hand Telerehabilitation Intervention in an Adolescent With Hemiplegic Cerebral Palsy.” Journal of Child Neurology 26, no. 3 (March 2011): 389–393. doi:10.1177/0883073810394847. |
| Gordon, C., S. Roopchand-Martin, and A. Gregg. “Potential of the Nintendo Wii™ as a Rehabilitation Tool for Children with Cerebral Palsy in a Developing Country: a Pilot Study.” Physiotherapy 98, no. 3 (September 2012): 238–242. doi:10.1016/j.physio.2012.05.011. |
| Howcroft, Jennifer, Darcy Fehlings, Virginia Wright, Karl Zabjek, Jan Andrysek, and Elaine Biddiss. “A Comparison of Solo and Multiplayer Active Videogame Play in Children with Unilateral Cerebral Palsy.” Games for Health Journal 1, no. 4 (August 2012): 287–293. doi:10.1089/g4h.2012.0015.* |
| Howcroft, Jennifer, Sue Klejman, Darcy Fehlings, Virginia Wright, Karl Zabjek, Jan Andrysek, and Elaine Biddiss. “Active Video Game Play in Children With Cerebral Palsy: Potential for Physical Activity Promotion and Rehabilitation Therapies.” Archives of Physical Medicine and Rehabilitation 93, no. 8 (August 2012): 1448–1456. doi:10.1016/j.apmr.2012.02.033.* |
| Huber, M., B. Rabin, C. Docan, G.C. Burdea, M. AbdelBaky, and M.R. Golomb. “Feasibility of Modified Remotely Monitored In-Home Gaming Technology for Improving Hand Function in Adolescents With Cerebral Palsy.” IEEE Transactions on Information Technology in Biomedicine 14, no. 2 (March 2010): 526–534. doi:10.1109/titb.2009.2038995. |
| Hung, Jen-Wen, Yao-Jen Chang, and Wen-Ying Han. “Game Technology to Increase Range of Motion for Adolescents with Cerebral Palsy: a Feasibility Study.” International Journal on Disability and Human Development 16, no. 3 (January 1, 2017). doi:10.1515/ijdhd-2016-0026. |
| Hurkmans, Henri L., Rita J. van den Berg-Emons, and Henk J. Stam. “Energy Expenditure in Adults With Cerebral Palsy Playing Wii Sports.” Archives of Physical Medicine and Rehabilitation 91, no. 10 (October 2010): 1577–1581. doi:10.1016/j.apmr.2010.07.216. |
| Jannink, Michiel J. A., Gelske J. van der Wilden, Dorine W. Navis, Gerben Visser, Jeanine Gussinklo, and Maarten Ijzerman. “A Low-Cost Video Game Applied for Training of Upper Extremity Function in Children with Cerebral Palsy: A Pilot Study.” CyberPsychology & Behavior 11, no. 1 (February 2008): 27–32. doi:10.1089/cpb.2007.0014. |
| Jaume-i-Capo, Antoni, Pau Martinez-Bueso, Biel Moya-Alcover, and Javier Varona. “Interactive Rehabilitation System for Improvement of Balance Therapies in People With Cerebral Palsy.” IEEE Transactions on Neural Systems and Rehabilitation Engineering 22, no. 2 (March 2014): 419–427. doi:10.1109/tnsre.2013.2279155.* |
| Jaume-i-Capó, Antoni, Pau Martínez-Bueso, Biel Moyà-Alcover, and Javier Varona. “Improving Vision-Based Motor Rehabilitation Interactive Systems for Users with Disabilities Using Mirror Feedback.” The Scientific World Journal 2014 (2014): 1–9. doi:10.1155/2014/964576. |
| Jelsma, Jennifer, Marieke Pronk, Gillian Ferguson, and Dorothee Jelsma-Smit. “The Effect of the Nintendo Wii Fit on Balance Control and Gross Motor Function of Children with Spastic Hemiplegic Cerebral Palsy.” Developmental Neurorehabilitation 16, no. 1 (October 3, 2012): 27–37. doi:10.3109/17518423.2012.711781. |
| Kai Chen, Yupeng Ren, Deborah Gaebler-Spira, and Li-Qun Zhang. “Home-Based Tele-Assisted Robotic Rehabilitation of Joint Impairments in Children with Cerebral Palsy.” 2014 36th Annual International Conference of the IEEE Engineering in Medicine and Biology Society (August 2014). doi:10.1109/embc.2014.6944819. |
| Kassee, Caroline, Carolyn Hunt, Michael W.R. Holmes, and Meghann Lloyd. “Home-Based Nintendo Wii Training to Improve Upper-Limb Function in Children Ages 7 to 12 with Spastic Hemiplegic Cerebral Palsy.” Journal of Pediatric Rehabilitation Medicine 10, no. 2 (June 28, 2017): 145–154. doi:10.3233/prm-170439.* |
| Keller, Jeffrey W., and Hubertus J.A. van Hedel. “Weight-Supported Training of the Upper Extremity in Children with Cerebral Palsy: a Motor Learning Study.” Journal of NeuroEngineering and Rehabilitation 14, no. 1 (August 30, 2017). doi:10.1186/s12984-017-0293-3. |
| Knights, Shannon, Nicholas Graham, Lauren Switzer, Hamilton Hernandez, Zi Ye, Briar Findlay, Wen Yan Xie, Virginia Wright, and Darcy Fehlings. “An Innovative Cycling Exergame to Promote Cardiovascular Fitness in Youth with Cerebral Palsy: A Brief Report.” Developmental Neurorehabilitation (June 20, 2014): 1–6. doi:10.3109/17518423.2014.923056. |
| Levac, Danielle, Anna McCormick, Mindy F. Levin, Marie Brien, Richard Mills, Elka Miller, and Heidi Sveistrup. “Active Video Gaming for Children with Cerebral Palsy: Does a Clinic-Based Virtual Reality Component Offer an Additive Benefit? A Pilot Study.” Physical & Occupational Therapy In Pediatrics 38, no. 1 (April 4, 2017): 74–87. doi:10.1080/01942638.2017.1287810.* |
| Li, W., Lam-Damji, S., Chau, T., & Fehlings, D. (2009). The development of a home-based virtual reality therapy system to promote upper extremity movement for children with hemiplegic cerebral palsy. Technology and Disability, 21(3), 107-113. |
| Liu, Ling, Xiang Chen, Zhiyuan Lu, Shuai Cao, De Wu, and Xu Zhang. “Development of an EMG-ACC-Based Upper Limb Rehabilitation Training System.” IEEE Transactions on Neural Systems and Rehabilitation Engineering 25, no. 3 (March 2017): 244–253. doi:10.1109/tnsre.2016.2560906.* |
| MacIntosh, Alexander, Lauren Switzer, Susan Hwang, Adrian L. Jessup Schneider, Daniel Clarke, T.C. Nicholas Graham, and Darcy L. Fehlings. “Ability-Based Balancing Using the Gross Motor Function Measure in Exergaming for Youth with Cerebral Palsy.” Games for Health Journal 6, no. 6 (December 2017): 379–385. doi:10.1089/g4h.2017.0053.* |
| MacIntosh, Alexander; Switzer, Lauren; Hernandez, Hamilton; Hwang, Susan; Schneider, Adrian L. Jessup; Moran, Daniel; Graham, T. C. Nicholas; Fehlings, Darcy L. “Balancing for Gross Motor Ability Differences in Exercise Videogames Between Youth with Cerebral Palsy at Gross Motor Function Classification System Levels II and III.” Developmental Medicine & Child Neurology 58 (September 2016): 110–111. doi:10.1111/dmcn.50_13225. |
| Meyns, Pieter, Liene Pans, Kaat Plasmans, Lieve Heyrman, Kaat Desloovere, and Guy Molenaers. “The Effect of Additional Virtual Reality Training on Balance in Children with Cerebral Palsy after Lower Limb Surgery: A Feasibility Study.” Games for Health Journal 6, no. 1 (February 2017): 39–48. doi:10.1089/g4h.2016.0069. |
| Nash, Janet, Peter D. Neilson, and Nicholas J. O’Dwyer. “REDUCING SPASTICITY TO CONTROL MUSCLE CONTRACTURE OF CHILDREN WITH CEREBRAL PALSY.” Developmental Medicine & Child Neurology 31, no. 4 (November 12, 2008): 471–480. doi:10.1111/j.1469-8749.1989.tb04025.x. |
| Ni, Lian Ting, Darcy Fehlings, and Elaine Biddiss. “Design and Evaluation of Virtual Reality–Based Therapy Games with Dual Focus on Therapeutic Relevance and User Experience for Children with Cerebral Palsy.” Games for Health Journal 3, no. 3 (June 2014): 162–171. doi:10.1089/g4h.2014.0003. |
| Odle, Brooke M., Amanda Irving, and Richard Foulds. “Usability of an Adaptable Video Game Platform for Children with Cerebral Palsy.” 2009 IEEE 35th Annual Northeast Bioengineering Conference (April 2009). doi:10.1109/nebc.2009.4967748. |
| Pavão, Silvia Leticia, Joice Luiza Bruno Arnoni, Alyne Kalyane Câmara de Oliveira, and Nelci Adriana Cicuto Ferreira Rocha. “Impacto de Intervenção Baseada Em Realidade Virtual Sobre o Desempenho Motor e Equilíbrio de Uma Criança Com Paralisia Cerebral: Estudo de Caso1.” Revista Paulista de Pediatria 32, no. 4 (December 2014): 389–394. doi:10.1016/j.rpped.2014.04.005. |
| Peper, C. (Lieke) E., Edwin C. P. Van Loon, Anke Van de Rijt, Annelie Salverda, and Annette A. van Kuijk. “Bimanual Training for Children with Cerebral Palsy: Exploring the Effects of Lissajous-Based Computer Gaming.” Developmental Neurorehabilitation 16, no. 4 (March 11, 2013): 255–265. doi:10.3109/17518423.2012.760116. |
| Preston, N., A. Weightman, J. Gallagher, M. Levesley, M. Mon-Williams, M. Clarke, and R. J. OConnor. “A pilot single-blind multicentre randomized controlled trial to evaluate the potential benefits of computer-assisted arm rehabilitation gaming technology on the arm function of children with spastic cerebral palsy.” Clinical Rehabilitation 30, no. 10 (September 13, 2015): 1004–1015. doi:10.1177/0269215515604699.* |
| Psychouli, Pavlina, and Colin R. Kennedy. “Modified Constraint-Induced Movement Therapy as a Home-Based Intervention for Children With Cerebral Palsy.” Pediatric Physical Therapy 28, no. 2 (2016): 154–160. doi:10.1097/pep.0000000000000227. |
| Qiu, Qinyin, Diego A Ramirez, Soha Saleh, Gerard G Fluet, Heta D Parikh, Donna Kelly, and Sergei V Adamovich. “The New Jersey Institute of Technology Robot-Assisted Virtual Rehabilitation (NJIT-RAVR) System for Children with Cerebral Palsy: a Feasibility Study.” Journal of NeuroEngineering and Rehabilitation 6, no. 1 (2009): 40. doi:10.1186/1743-0003-6-40. |
| Ramstrand, N., & Lygnegård, F. “Can balance in children with cerebral palsy improve through use of an activity promoting computer game?”. Technology and Health Care, 20 (2012):531-540. doi: 10.3233/THC-2012-0696.* |
| Reifenberg, Grace, Gabrielle Gabrosek, Kelly Tanner, Karen Harpster, Rachel Proffitt, and Andrew Persch. “Feasibility of Pediatric Game-Based Neurorehabilitation Using Telehealth Technologies: A Case Report.” American Journal of Occupational Therapy 71, no. 3 (March 27, 2017): 7103190040p1. doi:10.5014/ajot.2017.024976. |
| Rios, D. C., T. Gilbertson, S. W. McCoy, R. Price, K. Gutman, K. E. F. Miller, A. Fechko, and C. T. Moritz. “NeuroGame Therapy to Improve Wrist Control in Children with Cerebral Palsy: A Case Series.” Developmental Neurorehabilitation 16, no. 6 (April 25, 2013): 398–409. doi:10.3109/17518423.2013.766818. |
| Robert, M., L. Ballaz, R. Hart, and M. Lemay. “Exercise Intensity Levels in Children With Cerebral Palsy While Playing With an Active Video Game Console.” Physical Therapy 93, no. 8 (April 11, 2013): 1084–1091. doi:10.2522/ptj.20120204. |
| Robert, Maxime T., Rhona Guberek, Mindy F. Levin, and Heidi Sveistrup. “Motor Learning of the Upper Limb in Children with Cerebral Palsy after Virtual and Physical Training Intervention.” 2013 International Conference on Virtual Rehabilitation (ICVR) (August 2013). doi:10.1109/icvr.2013.6662125. |
| Robert, Maxime T, and Mindy F Levin. “Validation of Reaching in a Virtual Environment in Typically Developing Children and Children with Mild Unilateral Cerebral Palsy.” Developmental Medicine & Child Neurology 60, no. 4 (February 10, 2018): 382–390. doi:10.1111/dmcn.13688. |
| Rowland, Jennifer L., and James H. Rimmer. “Feasibility of Using Active Video Gaming as a Means for Increasing Energy Expenditure in Three Nonambulatory Young Adults With Disabilities.” PM&R 4, no. 8 (August 2012): 569–573. doi:10.1016/j.pmrj.2012.03.011. |
| Sajan, Jane Elizabeth, Judy Ann John, Pearlin Grace, Sneha Sara Sabu, and George Tharion. “Wii-Based Interactive Video Games as a Supplement to Conventional Therapy for Rehabilitation of Children with Cerebral Palsy: A Pilot, Randomized Controlled Trial.” Developmental Neurorehabilitation 20, no. 6 (November 15, 2016): 361–367. doi:10.1080/17518423.2016.1252970. |
| Sandlund, Marlene, Eva Lindh Waterworth, Suzanne McDonough, and Charlotte Hager Ross. “Interactive Games in Motor Rehabilitation for Children with Sensorimotor Disorders.” 2007 Virtual Rehabilitation (September 2007). doi:10.1109/icvr.2007.4362136. |
| Sandlund, Marlene, Eva Lindh Waterworth, and Charlotte Häger. “Using Motion Interactive Games to Promote Physical Activity and Enhance Motor Performance in Children with Cerebral Palsy.” Developmental Neurorehabilitation 14, no. 1 (January 17, 2011): 15–21. doi:10.3109/17518423.2010.533329.* |
| Sevick, Marisa, Elizabeth Eklund, Allison Mensch, Matthew Foreman, John Standeven, and Jack Engsberg. “Using Free Internet Videogames in Upper Extremity Motor Training for Children with Cerebral Palsy.” Behavioral Sciences 6, no. 2 (June 7, 2016): 10. doi:10.3390/bs6020010.* |
| Sgandurra, Giuseppina, Adriano Ferrari, Giuseppe Cossu, Andrea Guzzetta, Leonardo Fogassi, and Giovanni Cioni. “Randomized Trial of Observation and Execution of Upper Extremity Actions Versus Action Alone in Children With Unilateral Cerebral Palsy.” Neurorehabilitation and Neural Repair 27, no. 9 (July 25, 2013): 808–815. doi:10.1177/1545968313497101. |
| Silva Dias, T., da Conceição, K. F., de Oliveira, A. I. A., & da Silva, R. L. M. The contributions of game therapy concerning motor performance of individual with cerebral palsy. 2017. Brazilian Journal of Occupational Therapy/Cadernos Brasileiros de Terapia Ocupacional, 25(3). |
| Stansfield, S., Dennis, C., Larin, H., & Gallagher, C. Movement-Based VR Gameplay Therapy For A Child With Cerebral Palsy. Annual Review of Cybertherapy and Telemedicine 2015. 2016. Virtual Reality in Healthcare: Medical Simulation and Experiential Interface, 219, 153. |
| Tarakci, Devrim, Burcu Ersoz Huseyinsinoglu, Ela Tarakci, and Arzu Razak Ozdincler. “Effects of Nintendo Wii-Fit®video Games on Balance in Children with Mild Cerebral Palsy.” Pediatrics International 58, no. 10 (August 23, 2016): 1042–1050. doi:10.1111/ped.12942. |
| Van Hedel, Hubertus J.A., Karin Wick, Andreas Meyer-Heim, and Kynan Eng. “Improving Dexterity in Children with Cerebral Palsy.” 2011 International Conference on Virtual Rehabilitation (June 2011). doi:10.1109/icvr.2011.5971872. |
| Velasco, Miguel A., Rafael Raya, Luca Muzzioli, Daniela Morelli, Abraham Otero, Marco Iosa, Febo Cincotti, and Eduardo Rocon. “Evaluation of Cervical Posture Improvement of Children with Cerebral Palsy after Physical Therapy Based on Head Movements and Serious Games.” BioMedical Engineering OnLine 16, no. S1 (August 2017). doi:10.1186/s12938-017-0364-5. |
| Wade, Will, and David Porter. “Sitting Playfully: Does the Use of a Centre of Gravity Computer Game Controller Influence the Sitting Ability of Young People with Cerebral Palsy?” Disability and Rehabilitation: Assistive Technology 7, no. 2 (October 4, 2011): 122–129. doi:10.3109/17483107.2011.589485. |
| Weightman, Andrew Patrick Hayes, Nick Preston, Raymond Holt, Matthew Allsop, Martin Levesley, and Bipinchandra Bhakta. “Engaging Children in Healthcare Technology Design: Developing Rehabilitation Technology for Children with Cerebral Palsy.” Journal of Engineering Design 21, no. 5 (March 25, 2009): 579–600. doi:10.1080/09544820802441092. |
| Winkels, Diny G. M., Anke I. R. Kottink, Rutger A. J. Temmink, Juliëtte M. M. Nijlant, and Jaap H. Buurke. “Wii™-Habilitation of Upper Extremity Function in Children with Cerebral Palsy. An Explorative Study.” Developmental Neurorehabilitation 16, no. 1 (October 3, 2012): 44–51. doi:10.3109/17518423.2012.713401.* |
| Yagüe Sebastián, M.P., M.M. Yagüe Sebastián, A. Lekuona Amiano, and M.C. Sanz Rubio. “Los Videojuegos En El Tratamiento Fisioterápico de La Parálisis Cerebral.” Fisioterapia 38, no. 6 (November 2016): 295–302. doi:10.1016/j.ft.2015.11.005.* |
| Yalon-Chamovitz, Shira, and Patrice L. (Tamar) Weiss. “Virtual Reality as a Leisure Activity for Young Adults with Physical and Intellectual Disabilities.” Research in Developmental Disabilities 29, no. 3 (May 2008): 273–287. doi:10.1016/j.ridd.2007.05.004.* |
| Yong, C. H., Wei, R. F. M., Aimei, M. K., Yanting, P., Shan, C. P., Leng, M. N. Y., & Kumar, D. S. Effects of virtual reality games with physiotherapy on balance of children with cerebral palsy. In Proceedings of the 5th International Conference on Rehabilitation Engineering & Assistive Technology. (2011, July) (p. 54). Singapore Therapeutic, Assistive & Rehabilitative Technologies (START) Centre. |
| Yoo, J. W., Lee, D. R., Sim, Y. J., You, J. H., & Kim, C. J. Effects of innovative virtual reality game and EMG biofeedback on neuromotor control in cerebral palsy. Bio-medical materials and engineering. 2014. 24(6): 3613-3618. doi: 10.3233/BME-141188. |
| Zoccolillo, L., Morelli, D., Cincotti, F., Muzzioli, L., Gobbetti, T., Paolucci, S., & Iosa, M. Video-game based therapy performed by children with cerebral palsy: a cross-over randomized controlled trial and a cross-sectional quantitative measure of physical activity. 2015. Eur J Phys Rehabil Med, 51(6), 669-76. |
| Zondervan, Daniel K., Riccardo Secoli, Aurelia Mclaughlin Darling, John Farris, Jan Furumasu, and David J. Reinkensmeyer. “Design and Evaluation of the Kinect-Wheelchair Interface Controlled (KWIC) Smart Wheelchair for Pediatric Powered Mobility Training.” Assistive Technology 27, no. 3 (May 26, 2015): 183–192. doi:10.1080/10400435.2015.1012607. |

| Alegre, M.I. Diez, and R. Cano de la Cuerda. “Empleo de un video juego como herramienta terapéutica en adultos con parálisis cerebral tipo tetraparesia espástica. estudio piloto.” Fisioterapia 34, no. 1 (January 2012): 23–30. doi:10.1016/j.ft.2011.09.001.* |
| --- |
| Atasavun Uysal, Songül, and Gül Baltaci. “Effects of Nintendo Wii™ Training on Occupational Performance, Balance, and Daily Living Activities in Children with Spastic Hemiplegic Cerebral Palsy: A Single-Blind and Randomized Trial.” Games for Health Journal 5, no. 5 (October 2016): 311–317. doi:10.1089/g4h.2015.0102. |
| Barton, Gabor J., Malcolm B. Hawken, Gill Holmes, and Michael H. Schwartz. “A Gait Index May Underestimate Changes of Gait: a Comparison of the Movement Deviation Profile and the Gait Deviation Index.” Computer Methods in Biomechanics and Biomedical Engineering 18, no. 1 (March 22, 2013): 57–63. doi:10.1080/10255842.2013.776549. |
| Bhakta, B, A Weightman, N Preston, M Levesley, R Holt, M Mon-Williams, M Clarke, and AJ Cozens. “Home Based Computer-Assisted Upper Limb Exercise for Young Children with Cerebral Palsy: A Feasibility Study Investigating Impact on Motor Control and Functional Outcome.” Journal of Rehabilitation Medicine 43, no. 4 (2011): 359–363. doi:10.2340/16501977-0679. |
| Bingham, Peter M., and Barbara Calhoun. “Digital Posturography Games Correlate with Gross Motor Function in Children with Cerebral Palsy.” Games for Health Journal 4, no. 2 (April 2015): 145–148. doi:10.1089/g4h.2014.0096. |
| Bonnechère, Bruno, Lubos Omelina, Bart Jansen, and Serge Van Sint Jan. “Balance Improvement after Physical Therapy Training Using Specially Developed Serious Games for Cerebral Palsy Children: Preliminary Results.” Disability and Rehabilitation 39, no. 4 (August 3, 2015): 403–406. doi:10.3109/09638288.2015.1073373. |
| Bonnechère, Bruno, Lubos Omelina, Bart Jansen, Marcel Rooze, and Serge Van Sint Jan. “Balance Training Using Specially Developed Serious Games for Cerebral Palsy Children, a Feasibility Study.” Proceedings of the 8th International Conference on Pervasive Computing Technologies for Healthcare (2014). doi:10.4108/icst.pervasivehealth.2014.255332. |
| Bulea, Thomas C., Zachary F. Lerner, Andrew J. Gravunder, and Diane L. Damiano. “Exergaming with a Pediatric Exoskeleton: Facilitating Rehabilitation and Research in Children with Cerebral Palsy.” 2017 International Conference on Rehabilitation Robotics (ICORR) (July 2017). doi:10.1109/icorr.2017.8009394. |
| Burdea, G. C., A. Jain, B. Rabin, R. Pellosie, and M. Golomb. “Long-Term Hand Tele-Rehabilitation on the Playstation 3: Benefits and Challenges.” 2011 Annual International Conference of the IEEE Engineering in Medicine and Biology Society (August 2011). doi:10.1109/iembs.2011.6090522. |
| Burdea, Grigore C., Daniel Cioi, Angad Kale, William E. Janes, Sandy A. Ross, and Jack R. Engsberg. “Robotics and Gaming to Improve Ankle Strength, Motor Control, and Function in Children With Cerebral Palsy—A Case Study Series.” IEEE Transactions on Neural Systems and Rehabilitation Engineering 21, no. 2 (March 2013): 165–173. doi:10.1109/tnsre.2012.2206055.* |
| Camara Machado, Fabiana Rita, Priscilla Pereira Antunes, Jandara De Moura Souza, Antônio Cardoso Dos Santos, Daniela Centenaro Levandowski, and Alcyr Alves De Oliveira. “Motor Improvement Using Motion Sensing Game Devices for Cerebral Palsy Rehabilitation.” Journal of Motor Behavior 49, no. 3 (September 3, 2016): 273–280. doi:10.1080/00222895.2016.1191422. |
| Chang, Yao-Jen, Wen-Ying Han, and Yu-Chi Tsai. “A Kinect-Based Upper Limb Rehabilitation System to Assist People with Cerebral Palsy.” Research in Developmental Disabilities 34, no. 11 (November 2013): 3654–3659. doi:10.1016/j.ridd.2013.08.021. |
| Chen, Kai, Yi-Ning Wu, Yupeng Ren, Lin Liu, Deborah Gaebler-Spira, Kelly Tankard, Julia Lee, Weiqun Song, Maobin Wang, and Li-Qun Zhang. “Home-Based Versus Laboratory-Based Robotic Ankle Training for Children With Cerebral Palsy: A Pilot Randomized Comparative Trial.” Archives of Physical Medicine and Rehabilitation 97, no. 8 (August 2016): 1237–1243. doi:10.1016/j.apmr.2016.01.029. |
| Chien-yu Lin, and Shu-hua Chen. “Effectiveness of Interactive Teaching Materials on Special Education and Assistive Technology.” 2014 9th International Conference on Computer Science & Education (August 2014). doi:10.1109/iccse.2014.6926479. |
| Chiu, Hsiu-Ching, Louise Ada, and Hsin-Min Lee. “Upper Limb Training Using Wii Sports Resort™ for Children with Hemiplegic Cerebral Palsy: a Randomized, Single-Blind Trial.” Clinical Rehabilitation 28, no. 10 (May 21, 2014): 1015–1024. doi:10.1177/0269215514533709. |
| Chung, Peter J., Douglas L. Vanderbilt, Sheree M. Schrager, Eugene Nguyen, and Eileen Fowler. “Active Videogaming for Individuals with Severe Movement Disorders: Results from a Community Study.” Games for Health Journal 4, no. 3 (June 2015): 190–194. doi:10.1089/g4h.2014.0091. |
| Curtis, Derek John, Jesper Bencke, and Bente Mygind. “The Effect of Training in an Interactive Dynamic Stander on Ankle Dorsiflexion and Gross Motor Function in Children with Cerebral Palsy.” Developmental Neurorehabilitation 17, no. 6 (May 27, 2014): 393–397. doi:10.3109/17518423.2013.844738. |
| Deutsch, J. E, M. Borbely, J. Filler, K. Huhn, and P. Guarrera-Bowlby. “Use of a Low-Cost, Commercially Available Gaming Console (Wii) for Rehabilitation of an Adolescent With Cerebral Palsy.” Physical Therapy 88, no. 10 (August 8, 2008): 1196–1207. doi:10.2522/ptj.20080062. |
| Do, Ji-Hye, Eun-Young Yoo, Min-Ye Jung, and Hae Yean Park. “The Effects of Virtual Reality-Based Bilateral Arm Training on Hemiplegic Children’s Upper Limb Motor Skills.” NeuroRehabilitation 38, no. 2 (February 18, 2016): 115–127. doi:10.3233/nre-161302. |
| Gallagher, Justin, Nick Preston, Raymond Holt, Mark Mon-Williams, Martin Levesley, and Andrew Weightman. “Assessment of Upper Limb Movement with an Autonomous Robotic Device in a School Environment for Children with Cerebral Palsy.” 2015 IEEE International Conference on Rehabilitation Robotics (ICORR) (August 2015). doi:10.1109/icorr.2015.7281295. |
| Gatica-Rojas, V., Méndez-Rebolledo, G., Guzman-Muñoz, E., Soto-Poblete, A., Cartes-Velásquez, R., Elgueta-Cancino, E., & Cofré, L. L. “Does Nintendo Wii Balance Board improve standing balance? A randomized controlled trial in children with cerebral palsy”. 2017. European journal of physical and rehabilitation medicine, 53(4): 535-544. doi: 10.23736/S1973-9087.16.04447-6 |
| Gatica-Rojas, Valeska, Ricardo Cartes-Velásquez, Guillermo Méndez-Rebolledo, Eduardo Guzman-Muñoz, and L. Eduardo Cofré Lizama. “Effects of a Nintendo Wii Exercise Program on Spasticity and Static Standing Balance in Spastic Cerebral Palsy.” Developmental Neurorehabilitation 20, no. 6 (August 18, 2016): 388–391. doi:10.1080/17518423.2016.1211770. |
| Gerber, Corinna N., Bettina Kunz, and Hubertus J. A. van Hedel. “Preparing a Neuropediatric Upper Limb Exergame Rehabilitation System for Home-Use: a Feasibility Study.” Journal of NeuroEngineering and Rehabilitation 13, no. 1 (March 23, 2016). doi:10.1186/s12984-016-0141-x. |
| Golomb, Meredith R., Brenna C. McDonald, Stuart J. Warden, Janell Yonkman, Andrew J. Saykin, Bridget Shirley, Meghan Huber, et al. “In-Home Virtual Reality Videogame Telerehabilitation in Adolescents With Hemiplegic Cerebral Palsy.” Archives of Physical Medicine and Rehabilitation 91, no. 1 (January 2010): 1–8.e1. doi:10.1016/j.apmr.2009.08.153. |
| Golomb, Meredith R., Stuart J. Warden, Elaine Fess, Bryan Rabin, Janell Yonkman, Bridget Shirley, and Grigore C. Burdea. “Maintained Hand Function and Forearm Bone Health 14 Months After an In-Home Virtual-Reality Videogame Hand Telerehabilitation Intervention in an Adolescent With Hemiplegic Cerebral Palsy.” Journal of Child Neurology 26, no. 3 (March 2011): 389–393. doi:10.1177/0883073810394847. |
| Gordon, C., S. Roopchand-Martin, and A. Gregg. “Potential of the Nintendo Wii™ as a Rehabilitation Tool for Children with Cerebral Palsy in a Developing Country: a Pilot Study.” Physiotherapy 98, no. 3 (September 2012): 238–242. doi:10.1016/j.physio.2012.05.011. |
| Howcroft, Jennifer, Darcy Fehlings, Virginia Wright, Karl Zabjek, Jan Andrysek, and Elaine Biddiss. “A Comparison of Solo and Multiplayer Active Videogame Play in Children with Unilateral Cerebral Palsy.” Games for Health Journal 1, no. 4 (August 2012): 287–293. doi:10.1089/g4h.2012.0015.* |
| Howcroft, Jennifer, Sue Klejman, Darcy Fehlings, Virginia Wright, Karl Zabjek, Jan Andrysek, and Elaine Biddiss. “Active Video Game Play in Children With Cerebral Palsy: Potential for Physical Activity Promotion and Rehabilitation Therapies.” Archives of Physical Medicine and Rehabilitation 93, no. 8 (August 2012): 1448–1456. doi:10.1016/j.apmr.2012.02.033.* |
| Huber, M., B. Rabin, C. Docan, G.C. Burdea, M. AbdelBaky, and M.R. Golomb. “Feasibility of Modified Remotely Monitored In-Home Gaming Technology for Improving Hand Function in Adolescents With Cerebral Palsy.” IEEE Transactions on Information Technology in Biomedicine 14, no. 2 (March 2010): 526–534. doi:10.1109/titb.2009.2038995. |
| Hung, Jen-Wen, Yao-Jen Chang, and Wen-Ying Han. “Game Technology to Increase Range of Motion for Adolescents with Cerebral Palsy: a Feasibility Study.” International Journal on Disability and Human Development 16, no. 3 (January 1, 2017). doi:10.1515/ijdhd-2016-0026. |
| Hurkmans, Henri L., Rita J. van den Berg-Emons, and Henk J. Stam. “Energy Expenditure in Adults With Cerebral Palsy Playing Wii Sports.” Archives of Physical Medicine and Rehabilitation 91, no. 10 (October 2010): 1577–1581. doi:10.1016/j.apmr.2010.07.216. |
| Jannink, Michiel J. A., Gelske J. van der Wilden, Dorine W. Navis, Gerben Visser, Jeanine Gussinklo, and Maarten Ijzerman. “A Low-Cost Video Game Applied for Training of Upper Extremity Function in Children with Cerebral Palsy: A Pilot Study.” CyberPsychology & Behavior 11, no. 1 (February 2008): 27–32. doi:10.1089/cpb.2007.0014. |
| Jaume-i-Capo, Antoni, Pau Martinez-Bueso, Biel Moya-Alcover, and Javier Varona. “Interactive Rehabilitation System for Improvement of Balance Therapies in People With Cerebral Palsy.” IEEE Transactions on Neural Systems and Rehabilitation Engineering 22, no. 2 (March 2014): 419–427. doi:10.1109/tnsre.2013.2279155.* |
| Jaume-i-Capó, Antoni, Pau Martínez-Bueso, Biel Moyà-Alcover, and Javier Varona. “Improving Vision-Based Motor Rehabilitation Interactive Systems for Users with Disabilities Using Mirror Feedback.” The Scientific World Journal 2014 (2014): 1–9. doi:10.1155/2014/964576. |
| Jelsma, Jennifer, Marieke Pronk, Gillian Ferguson, and Dorothee Jelsma-Smit. “The Effect of the Nintendo Wii Fit on Balance Control and Gross Motor Function of Children with Spastic Hemiplegic Cerebral Palsy.” Developmental Neurorehabilitation 16, no. 1 (October 3, 2012): 27–37. doi:10.3109/17518423.2012.711781. |
| Kai Chen, Yupeng Ren, Deborah Gaebler-Spira, and Li-Qun Zhang. “Home-Based Tele-Assisted Robotic Rehabilitation of Joint Impairments in Children with Cerebral Palsy.” 2014 36th Annual International Conference of the IEEE Engineering in Medicine and Biology Society (August 2014). doi:10.1109/embc.2014.6944819. |
| Kassee, Caroline, Carolyn Hunt, Michael W.R. Holmes, and Meghann Lloyd. “Home-Based Nintendo Wii Training to Improve Upper-Limb Function in Children Ages 7 to 12 with Spastic Hemiplegic Cerebral Palsy.” Journal of Pediatric Rehabilitation Medicine 10, no. 2 (June 28, 2017): 145–154. doi:10.3233/prm-170439.* |
| Keller, Jeffrey W., and Hubertus J.A. van Hedel. “Weight-Supported Training of the Upper Extremity in Children with Cerebral Palsy: a Motor Learning Study.” Journal of NeuroEngineering and Rehabilitation 14, no. 1 (August 30, 2017). doi:10.1186/s12984-017-0293-3. |
| Knights, Shannon, Nicholas Graham, Lauren Switzer, Hamilton Hernandez, Zi Ye, Briar Findlay, Wen Yan Xie, Virginia Wright, and Darcy Fehlings. “An Innovative Cycling Exergame to Promote Cardiovascular Fitness in Youth with Cerebral Palsy: A Brief Report.” Developmental Neurorehabilitation (June 20, 2014): 1–6. doi:10.3109/17518423.2014.923056. |
| Levac, Danielle, Anna McCormick, Mindy F. Levin, Marie Brien, Richard Mills, Elka Miller, and Heidi Sveistrup. “Active Video Gaming for Children with Cerebral Palsy: Does a Clinic-Based Virtual Reality Component Offer an Additive Benefit? A Pilot Study.” Physical & Occupational Therapy In Pediatrics 38, no. 1 (April 4, 2017): 74–87. doi:10.1080/01942638.2017.1287810.* |
| Li, W., Lam-Damji, S., Chau, T., & Fehlings, D. (2009). The development of a home-based virtual reality therapy system to promote upper extremity movement for children with hemiplegic cerebral palsy. Technology and Disability, 21(3), 107-113. |
| Liu, Ling, Xiang Chen, Zhiyuan Lu, Shuai Cao, De Wu, and Xu Zhang. “Development of an EMG-ACC-Based Upper Limb Rehabilitation Training System.” IEEE Transactions on Neural Systems and Rehabilitation Engineering 25, no. 3 (March 2017): 244–253. doi:10.1109/tnsre.2016.2560906.* |
| MacIntosh, Alexander, Lauren Switzer, Susan Hwang, Adrian L. Jessup Schneider, Daniel Clarke, T.C. Nicholas Graham, and Darcy L. Fehlings. “Ability-Based Balancing Using the Gross Motor Function Measure in Exergaming for Youth with Cerebral Palsy.” Games for Health Journal 6, no. 6 (December 2017): 379–385. doi:10.1089/g4h.2017.0053.* |
| MacIntosh, Alexander; Switzer, Lauren; Hernandez, Hamilton; Hwang, Susan; Schneider, Adrian L. Jessup; Moran, Daniel; Graham, T. C. Nicholas; Fehlings, Darcy L. “Balancing for Gross Motor Ability Differences in Exercise Videogames Between Youth with Cerebral Palsy at Gross Motor Function Classification System Levels II and III.” Developmental Medicine & Child Neurology 58 (September 2016): 110–111. doi:10.1111/dmcn.50_13225. |
| Meyns, Pieter, Liene Pans, Kaat Plasmans, Lieve Heyrman, Kaat Desloovere, and Guy Molenaers. “The Effect of Additional Virtual Reality Training on Balance in Children with Cerebral Palsy after Lower Limb Surgery: A Feasibility Study.” Games for Health Journal 6, no. 1 (February 2017): 39–48. doi:10.1089/g4h.2016.0069. |
| Nash, Janet, Peter D. Neilson, and Nicholas J. O’Dwyer. “REDUCING SPASTICITY TO CONTROL MUSCLE CONTRACTURE OF CHILDREN WITH CEREBRAL PALSY.” Developmental Medicine & Child Neurology 31, no. 4 (November 12, 2008): 471–480. doi:10.1111/j.1469-8749.1989.tb04025.x. |
| Ni, Lian Ting, Darcy Fehlings, and Elaine Biddiss. “Design and Evaluation of Virtual Reality–Based Therapy Games with Dual Focus on Therapeutic Relevance and User Experience for Children with Cerebral Palsy.” Games for Health Journal 3, no. 3 (June 2014): 162–171. doi:10.1089/g4h.2014.0003. |
| Odle, Brooke M., Amanda Irving, and Richard Foulds. “Usability of an Adaptable Video Game Platform for Children with Cerebral Palsy.” 2009 IEEE 35th Annual Northeast Bioengineering Conference (April 2009). doi:10.1109/nebc.2009.4967748. |
| Pavão, Silvia Leticia, Joice Luiza Bruno Arnoni, Alyne Kalyane Câmara de Oliveira, and Nelci Adriana Cicuto Ferreira Rocha. “Impacto de Intervenção Baseada Em Realidade Virtual Sobre o Desempenho Motor e Equilíbrio de Uma Criança Com Paralisia Cerebral: Estudo de Caso1.” Revista Paulista de Pediatria 32, no. 4 (December 2014): 389–394. doi:10.1016/j.rpped.2014.04.005. |
| Peper, C. (Lieke) E., Edwin C. P. Van Loon, Anke Van de Rijt, Annelie Salverda, and Annette A. van Kuijk. “Bimanual Training for Children with Cerebral Palsy: Exploring the Effects of Lissajous-Based Computer Gaming.” Developmental Neurorehabilitation 16, no. 4 (March 11, 2013): 255–265. doi:10.3109/17518423.2012.760116. |
| Preston, N., A. Weightman, J. Gallagher, M. Levesley, M. Mon-Williams, M. Clarke, and R. J. OConnor. “A pilot single-blind multicentre randomized controlled trial to evaluate the potential benefits of computer-assisted arm rehabilitation gaming technology on the arm function of children with spastic cerebral palsy.” Clinical Rehabilitation 30, no. 10 (September 13, 2015): 1004–1015. doi:10.1177/0269215515604699.* |
| Psychouli, Pavlina, and Colin R. Kennedy. “Modified Constraint-Induced Movement Therapy as a Home-Based Intervention for Children With Cerebral Palsy.” Pediatric Physical Therapy 28, no. 2 (2016): 154–160. doi:10.1097/pep.0000000000000227. |
| Qiu, Qinyin, Diego A Ramirez, Soha Saleh, Gerard G Fluet, Heta D Parikh, Donna Kelly, and Sergei V Adamovich. “The New Jersey Institute of Technology Robot-Assisted Virtual Rehabilitation (NJIT-RAVR) System for Children with Cerebral Palsy: a Feasibility Study.” Journal of NeuroEngineering and Rehabilitation 6, no. 1 (2009): 40. doi:10.1186/1743-0003-6-40. |
| Ramstrand, N., & Lygnegård, F. “Can balance in children with cerebral palsy improve through use of an activity promoting computer game?”. Technology and Health Care, 20 (2012):531-540. doi: 10.3233/THC-2012-0696.* |
| Reifenberg, Grace, Gabrielle Gabrosek, Kelly Tanner, Karen Harpster, Rachel Proffitt, and Andrew Persch. “Feasibility of Pediatric Game-Based Neurorehabilitation Using Telehealth Technologies: A Case Report.” American Journal of Occupational Therapy 71, no. 3 (March 27, 2017): 7103190040p1. doi:10.5014/ajot.2017.024976. |
| Rios, D. C., T. Gilbertson, S. W. McCoy, R. Price, K. Gutman, K. E. F. Miller, A. Fechko, and C. T. Moritz. “NeuroGame Therapy to Improve Wrist Control in Children with Cerebral Palsy: A Case Series.” Developmental Neurorehabilitation 16, no. 6 (April 25, 2013): 398–409. doi:10.3109/17518423.2013.766818. |
| Robert, M., L. Ballaz, R. Hart, and M. Lemay. “Exercise Intensity Levels in Children With Cerebral Palsy While Playing With an Active Video Game Console.” Physical Therapy 93, no. 8 (April 11, 2013): 1084–1091. doi:10.2522/ptj.20120204. |
| Robert, Maxime T., Rhona Guberek, Mindy F. Levin, and Heidi Sveistrup. “Motor Learning of the Upper Limb in Children with Cerebral Palsy after Virtual and Physical Training Intervention.” 2013 International Conference on Virtual Rehabilitation (ICVR) (August 2013). doi:10.1109/icvr.2013.6662125. |
| Robert, Maxime T, and Mindy F Levin. “Validation of Reaching in a Virtual Environment in Typically Developing Children and Children with Mild Unilateral Cerebral Palsy.” Developmental Medicine & Child Neurology 60, no. 4 (February 10, 2018): 382–390. doi:10.1111/dmcn.13688. |
| Rowland, Jennifer L., and James H. Rimmer. “Feasibility of Using Active Video Gaming as a Means for Increasing Energy Expenditure in Three Nonambulatory Young Adults With Disabilities.” PM&R 4, no. 8 (August 2012): 569–573. doi:10.1016/j.pmrj.2012.03.011. |
| Sajan, Jane Elizabeth, Judy Ann John, Pearlin Grace, Sneha Sara Sabu, and George Tharion. “Wii-Based Interactive Video Games as a Supplement to Conventional Therapy for Rehabilitation of Children with Cerebral Palsy: A Pilot, Randomized Controlled Trial.” Developmental Neurorehabilitation 20, no. 6 (November 15, 2016): 361–367. doi:10.1080/17518423.2016.1252970. |
| Sandlund, Marlene, Eva Lindh Waterworth, Suzanne McDonough, and Charlotte Hager Ross. “Interactive Games in Motor Rehabilitation for Children with Sensorimotor Disorders.” 2007 Virtual Rehabilitation (September 2007). doi:10.1109/icvr.2007.4362136. |
| Sandlund, Marlene, Eva Lindh Waterworth, and Charlotte Häger. “Using Motion Interactive Games to Promote Physical Activity and Enhance Motor Performance in Children with Cerebral Palsy.” Developmental Neurorehabilitation 14, no. 1 (January 17, 2011): 15–21. doi:10.3109/17518423.2010.533329.* |
| Sevick, Marisa, Elizabeth Eklund, Allison Mensch, Matthew Foreman, John Standeven, and Jack Engsberg. “Using Free Internet Videogames in Upper Extremity Motor Training for Children with Cerebral Palsy.” Behavioral Sciences 6, no. 2 (June 7, 2016): 10. doi:10.3390/bs6020010.* |
| Sgandurra, Giuseppina, Adriano Ferrari, Giuseppe Cossu, Andrea Guzzetta, Leonardo Fogassi, and Giovanni Cioni. “Randomized Trial of Observation and Execution of Upper Extremity Actions Versus Action Alone in Children With Unilateral Cerebral Palsy.” Neurorehabilitation and Neural Repair 27, no. 9 (July 25, 2013): 808–815. doi:10.1177/1545968313497101. |
| Silva Dias, T., da Conceição, K. F., de Oliveira, A. I. A., & da Silva, R. L. M. The contributions of game therapy concerning motor performance of individual with cerebral palsy. 2017. Brazilian Journal of Occupational Therapy/Cadernos Brasileiros de Terapia Ocupacional, 25(3). |
| Stansfield, S., Dennis, C., Larin, H., & Gallagher, C. Movement-Based VR Gameplay Therapy For A Child With Cerebral Palsy. Annual Review of Cybertherapy and Telemedicine 2015. 2016. Virtual Reality in Healthcare: Medical Simulation and Experiential Interface, 219, 153. |
| Tarakci, Devrim, Burcu Ersoz Huseyinsinoglu, Ela Tarakci, and Arzu Razak Ozdincler. “Effects of Nintendo Wii-Fit®video Games on Balance in Children with Mild Cerebral Palsy.” Pediatrics International 58, no. 10 (August 23, 2016): 1042–1050. doi:10.1111/ped.12942. |
| Van Hedel, Hubertus J.A., Karin Wick, Andreas Meyer-Heim, and Kynan Eng. “Improving Dexterity in Children with Cerebral Palsy.” 2011 International Conference on Virtual Rehabilitation (June 2011). doi:10.1109/icvr.2011.5971872. |
| Velasco, Miguel A., Rafael Raya, Luca Muzzioli, Daniela Morelli, Abraham Otero, Marco Iosa, Febo Cincotti, and Eduardo Rocon. “Evaluation of Cervical Posture Improvement of Children with Cerebral Palsy after Physical Therapy Based on Head Movements and Serious Games.” BioMedical Engineering OnLine 16, no. S1 (August 2017). doi:10.1186/s12938-017-0364-5. |
| Wade, Will, and David Porter. “Sitting Playfully: Does the Use of a Centre of Gravity Computer Game Controller Influence the Sitting Ability of Young People with Cerebral Palsy?” Disability and Rehabilitation: Assistive Technology 7, no. 2 (October 4, 2011): 122–129. doi:10.3109/17483107.2011.589485. |
| Weightman, Andrew Patrick Hayes, Nick Preston, Raymond Holt, Matthew Allsop, Martin Levesley, and Bipinchandra Bhakta. “Engaging Children in Healthcare Technology Design: Developing Rehabilitation Technology for Children with Cerebral Palsy.” Journal of Engineering Design 21, no. 5 (March 25, 2009): 579–600. doi:10.1080/09544820802441092. |
| Winkels, Diny G. M., Anke I. R. Kottink, Rutger A. J. Temmink, Juliëtte M. M. Nijlant, and Jaap H. Buurke. “Wii™-Habilitation of Upper Extremity Function in Children with Cerebral Palsy. An Explorative Study.” Developmental Neurorehabilitation 16, no. 1 (October 3, 2012): 44–51. doi:10.3109/17518423.2012.713401.* |
| Yagüe Sebastián, M.P., M.M. Yagüe Sebastián, A. Lekuona Amiano, and M.C. Sanz Rubio. “Los Videojuegos En El Tratamiento Fisioterápico de La Parálisis Cerebral.” Fisioterapia 38, no. 6 (November 2016): 295–302. doi:10.1016/j.ft.2015.11.005.* |
| Yalon-Chamovitz, Shira, and Patrice L. (Tamar) Weiss. “Virtual Reality as a Leisure Activity for Young Adults with Physical and Intellectual Disabilities.” Research in Developmental Disabilities 29, no. 3 (May 2008): 273–287. doi:10.1016/j.ridd.2007.05.004.* |
| Yong, C. H., Wei, R. F. M., Aimei, M. K., Yanting, P., Shan, C. P., Leng, M. N. Y., & Kumar, D. S. Effects of virtual reality games with physiotherapy on balance of children with cerebral palsy. In Proceedings of the 5th International Conference on Rehabilitation Engineering & Assistive Technology. (2011, July) (p. 54). Singapore Therapeutic, Assistive & Rehabilitative Technologies (START) Centre. |
| Yoo, J. W., Lee, D. R., Sim, Y. J., You, J. H., & Kim, C. J. Effects of innovative virtual reality game and EMG biofeedback on neuromotor control in cerebral palsy. Bio-medical materials and engineering. 2014. 24(6): 3613-3618. doi: 10.3233/BME-141188. |
| Zoccolillo, L., Morelli, D., Cincotti, F., Muzzioli, L., Gobbetti, T., Paolucci, S., & Iosa, M. Video-game based therapy performed by children with cerebral palsy: a cross-over randomized controlled trial and a cross-sectional quantitative measure of physical activity. 2015. Eur J Phys Rehabil Med, 51(6), 669-76. |
| Zondervan, Daniel K., Riccardo Secoli, Aurelia Mclaughlin Darling, John Farris, Jan Furumasu, and David J. Reinkensmeyer. “Design and Evaluation of the Kinect-Wheelchair Interface Controlled (KWIC) Smart Wheelchair for Pediatric Powered Mobility Training.” Assistive Technology 27, no. 3 (May 26, 2015): 183–192. doi:10.1080/10400435.2015.1012607. |

| Alegre, M.I. Diez, and R. Cano de la Cuerda. “Empleo de un video juego como herramienta terapéutica en adultos con parálisis cerebral tipo tetraparesia espástica. estudio piloto.” Fisioterapia 34, no. 1 (January 2012): 23–30. doi:10.1016/j.ft.2011.09.001.* |
| --- |
| Atasavun Uysal, Songül, and Gül Baltaci. “Effects of Nintendo Wii™ Training on Occupational Performance, Balance, and Daily Living Activities in Children with Spastic Hemiplegic Cerebral Palsy: A Single-Blind and Randomized Trial.” Games for Health Journal 5, no. 5 (October 2016): 311–317. doi:10.1089/g4h.2015.0102. |
| Barton, Gabor J., Malcolm B. Hawken, Gill Holmes, and Michael H. Schwartz. “A Gait Index May Underestimate Changes of Gait: a Comparison of the Movement Deviation Profile and the Gait Deviation Index.” Computer Methods in Biomechanics and Biomedical Engineering 18, no. 1 (March 22, 2013): 57–63. doi:10.1080/10255842.2013.776549. |
| Bhakta, B, A Weightman, N Preston, M Levesley, R Holt, M Mon-Williams, M Clarke, and AJ Cozens. “Home Based Computer-Assisted Upper Limb Exercise for Young Children with Cerebral Palsy: A Feasibility Study Investigating Impact on Motor Control and Functional Outcome.” Journal of Rehabilitation Medicine 43, no. 4 (2011): 359–363. doi:10.2340/16501977-0679. |
| Bingham, Peter M., and Barbara Calhoun. “Digital Posturography Games Correlate with Gross Motor Function in Children with Cerebral Palsy.” Games for Health Journal 4, no. 2 (April 2015): 145–148. doi:10.1089/g4h.2014.0096. |
| Bonnechère, Bruno, Lubos Omelina, Bart Jansen, and Serge Van Sint Jan. “Balance Improvement after Physical Therapy Training Using Specially Developed Serious Games for Cerebral Palsy Children: Preliminary Results.” Disability and Rehabilitation 39, no. 4 (August 3, 2015): 403–406. doi:10.3109/09638288.2015.1073373. |
| Bonnechère, Bruno, Lubos Omelina, Bart Jansen, Marcel Rooze, and Serge Van Sint Jan. “Balance Training Using Specially Developed Serious Games for Cerebral Palsy Children, a Feasibility Study.” Proceedings of the 8th International Conference on Pervasive Computing Technologies for Healthcare (2014). doi:10.4108/icst.pervasivehealth.2014.255332. |
| Bulea, Thomas C., Zachary F. Lerner, Andrew J. Gravunder, and Diane L. Damiano. “Exergaming with a Pediatric Exoskeleton: Facilitating Rehabilitation and Research in Children with Cerebral Palsy.” 2017 International Conference on Rehabilitation Robotics (ICORR) (July 2017). doi:10.1109/icorr.2017.8009394. |
| Burdea, G. C., A. Jain, B. Rabin, R. Pellosie, and M. Golomb. “Long-Term Hand Tele-Rehabilitation on the Playstation 3: Benefits and Challenges.” 2011 Annual International Conference of the IEEE Engineering in Medicine and Biology Society (August 2011). doi:10.1109/iembs.2011.6090522. |
| Burdea, Grigore C., Daniel Cioi, Angad Kale, William E. Janes, Sandy A. Ross, and Jack R. Engsberg. “Robotics and Gaming to Improve Ankle Strength, Motor Control, and Function in Children With Cerebral Palsy—A Case Study Series.” IEEE Transactions on Neural Systems and Rehabilitation Engineering 21, no. 2 (March 2013): 165–173. doi:10.1109/tnsre.2012.2206055.* |
| Camara Machado, Fabiana Rita, Priscilla Pereira Antunes, Jandara De Moura Souza, Antônio Cardoso Dos Santos, Daniela Centenaro Levandowski, and Alcyr Alves De Oliveira. “Motor Improvement Using Motion Sensing Game Devices for Cerebral Palsy Rehabilitation.” Journal of Motor Behavior 49, no. 3 (September 3, 2016): 273–280. doi:10.1080/00222895.2016.1191422. |
| Chang, Yao-Jen, Wen-Ying Han, and Yu-Chi Tsai. “A Kinect-Based Upper Limb Rehabilitation System to Assist People with Cerebral Palsy.” Research in Developmental Disabilities 34, no. 11 (November 2013): 3654–3659. doi:10.1016/j.ridd.2013.08.021. |
| Chen, Kai, Yi-Ning Wu, Yupeng Ren, Lin Liu, Deborah Gaebler-Spira, Kelly Tankard, Julia Lee, Weiqun Song, Maobin Wang, and Li-Qun Zhang. “Home-Based Versus Laboratory-Based Robotic Ankle Training for Children With Cerebral Palsy: A Pilot Randomized Comparative Trial.” Archives of Physical Medicine and Rehabilitation 97, no. 8 (August 2016): 1237–1243. doi:10.1016/j.apmr.2016.01.029. |
| Chien-yu Lin, and Shu-hua Chen. “Effectiveness of Interactive Teaching Materials on Special Education and Assistive Technology.” 2014 9th International Conference on Computer Science & Education (August 2014). doi:10.1109/iccse.2014.6926479. |
| Chiu, Hsiu-Ching, Louise Ada, and Hsin-Min Lee. “Upper Limb Training Using Wii Sports Resort™ for Children with Hemiplegic Cerebral Palsy: a Randomized, Single-Blind Trial.” Clinical Rehabilitation 28, no. 10 (May 21, 2014): 1015–1024. doi:10.1177/0269215514533709. |
| Chung, Peter J., Douglas L. Vanderbilt, Sheree M. Schrager, Eugene Nguyen, and Eileen Fowler. “Active Videogaming for Individuals with Severe Movement Disorders: Results from a Community Study.” Games for Health Journal 4, no. 3 (June 2015): 190–194. doi:10.1089/g4h.2014.0091. |
| Curtis, Derek John, Jesper Bencke, and Bente Mygind. “The Effect of Training in an Interactive Dynamic Stander on Ankle Dorsiflexion and Gross Motor Function in Children with Cerebral Palsy.” Developmental Neurorehabilitation 17, no. 6 (May 27, 2014): 393–397. doi:10.3109/17518423.2013.844738. |
| Deutsch, J. E, M. Borbely, J. Filler, K. Huhn, and P. Guarrera-Bowlby. “Use of a Low-Cost, Commercially Available Gaming Console (Wii) for Rehabilitation of an Adolescent With Cerebral Palsy.” Physical Therapy 88, no. 10 (August 8, 2008): 1196–1207. doi:10.2522/ptj.20080062. |
| Do, Ji-Hye, Eun-Young Yoo, Min-Ye Jung, and Hae Yean Park. “The Effects of Virtual Reality-Based Bilateral Arm Training on Hemiplegic Children’s Upper Limb Motor Skills.” NeuroRehabilitation 38, no. 2 (February 18, 2016): 115–127. doi:10.3233/nre-161302. |
| Gallagher, Justin, Nick Preston, Raymond Holt, Mark Mon-Williams, Martin Levesley, and Andrew Weightman. “Assessment of Upper Limb Movement with an Autonomous Robotic Device in a School Environment for Children with Cerebral Palsy.” 2015 IEEE International Conference on Rehabilitation Robotics (ICORR) (August 2015). doi:10.1109/icorr.2015.7281295. |
| Gatica-Rojas, V., Méndez-Rebolledo, G., Guzman-Muñoz, E., Soto-Poblete, A., Cartes-Velásquez, R., Elgueta-Cancino, E., & Cofré, L. L. “Does Nintendo Wii Balance Board improve standing balance? A randomized controlled trial in children with cerebral palsy”. 2017. European journal of physical and rehabilitation medicine, 53(4): 535-544. doi: 10.23736/S1973-9087.16.04447-6 |
| Gatica-Rojas, Valeska, Ricardo Cartes-Velásquez, Guillermo Méndez-Rebolledo, Eduardo Guzman-Muñoz, and L. Eduardo Cofré Lizama. “Effects of a Nintendo Wii Exercise Program on Spasticity and Static Standing Balance in Spastic Cerebral Palsy.” Developmental Neurorehabilitation 20, no. 6 (August 18, 2016): 388–391. doi:10.1080/17518423.2016.1211770. |
| Gerber, Corinna N., Bettina Kunz, and Hubertus J. A. van Hedel. “Preparing a Neuropediatric Upper Limb Exergame Rehabilitation System for Home-Use: a Feasibility Study.” Journal of NeuroEngineering and Rehabilitation 13, no. 1 (March 23, 2016). doi:10.1186/s12984-016-0141-x. |
| Golomb, Meredith R., Brenna C. McDonald, Stuart J. Warden, Janell Yonkman, Andrew J. Saykin, Bridget Shirley, Meghan Huber, et al. “In-Home Virtual Reality Videogame Telerehabilitation in Adolescents With Hemiplegic Cerebral Palsy.” Archives of Physical Medicine and Rehabilitation 91, no. 1 (January 2010): 1–8.e1. doi:10.1016/j.apmr.2009.08.153. |
| Golomb, Meredith R., Stuart J. Warden, Elaine Fess, Bryan Rabin, Janell Yonkman, Bridget Shirley, and Grigore C. Burdea. “Maintained Hand Function and Forearm Bone Health 14 Months After an In-Home Virtual-Reality Videogame Hand Telerehabilitation Intervention in an Adolescent With Hemiplegic Cerebral Palsy.” Journal of Child Neurology 26, no. 3 (March 2011): 389–393. doi:10.1177/0883073810394847. |
| Gordon, C., S. Roopchand-Martin, and A. Gregg. “Potential of the Nintendo Wii™ as a Rehabilitation Tool for Children with Cerebral Palsy in a Developing Country: a Pilot Study.” Physiotherapy 98, no. 3 (September 2012): 238–242. doi:10.1016/j.physio.2012.05.011. |
| Howcroft, Jennifer, Darcy Fehlings, Virginia Wright, Karl Zabjek, Jan Andrysek, and Elaine Biddiss. “A Comparison of Solo and Multiplayer Active Videogame Play in Children with Unilateral Cerebral Palsy.” Games for Health Journal 1, no. 4 (August 2012): 287–293. doi:10.1089/g4h.2012.0015.* |
| Howcroft, Jennifer, Sue Klejman, Darcy Fehlings, Virginia Wright, Karl Zabjek, Jan Andrysek, and Elaine Biddiss. “Active Video Game Play in Children With Cerebral Palsy: Potential for Physical Activity Promotion and Rehabilitation Therapies.” Archives of Physical Medicine and Rehabilitation 93, no. 8 (August 2012): 1448–1456. doi:10.1016/j.apmr.2012.02.033.* |
| Huber, M., B. Rabin, C. Docan, G.C. Burdea, M. AbdelBaky, and M.R. Golomb. “Feasibility of Modified Remotely Monitored In-Home Gaming Technology for Improving Hand Function in Adolescents With Cerebral Palsy.” IEEE Transactions on Information Technology in Biomedicine 14, no. 2 (March 2010): 526–534. doi:10.1109/titb.2009.2038995. |
| Hung, Jen-Wen, Yao-Jen Chang, and Wen-Ying Han. “Game Technology to Increase Range of Motion for Adolescents with Cerebral Palsy: a Feasibility Study.” International Journal on Disability and Human Development 16, no. 3 (January 1, 2017). doi:10.1515/ijdhd-2016-0026. |
| Hurkmans, Henri L., Rita J. van den Berg-Emons, and Henk J. Stam. “Energy Expenditure in Adults With Cerebral Palsy Playing Wii Sports.” Archives of Physical Medicine and Rehabilitation 91, no. 10 (October 2010): 1577–1581. doi:10.1016/j.apmr.2010.07.216. |
| Jannink, Michiel J. A., Gelske J. van der Wilden, Dorine W. Navis, Gerben Visser, Jeanine Gussinklo, and Maarten Ijzerman. “A Low-Cost Video Game Applied for Training of Upper Extremity Function in Children with Cerebral Palsy: A Pilot Study.” CyberPsychology & Behavior 11, no. 1 (February 2008): 27–32. doi:10.1089/cpb.2007.0014. |
| Jaume-i-Capo, Antoni, Pau Martinez-Bueso, Biel Moya-Alcover, and Javier Varona. “Interactive Rehabilitation System for Improvement of Balance Therapies in People With Cerebral Palsy.” IEEE Transactions on Neural Systems and Rehabilitation Engineering 22, no. 2 (March 2014): 419–427. doi:10.1109/tnsre.2013.2279155.* |
| Jaume-i-Capó, Antoni, Pau Martínez-Bueso, Biel Moyà-Alcover, and Javier Varona. “Improving Vision-Based Motor Rehabilitation Interactive Systems for Users with Disabilities Using Mirror Feedback.” The Scientific World Journal 2014 (2014): 1–9. doi:10.1155/2014/964576. |
| Jelsma, Jennifer, Marieke Pronk, Gillian Ferguson, and Dorothee Jelsma-Smit. “The Effect of the Nintendo Wii Fit on Balance Control and Gross Motor Function of Children with Spastic Hemiplegic Cerebral Palsy.” Developmental Neurorehabilitation 16, no. 1 (October 3, 2012): 27–37. doi:10.3109/17518423.2012.711781. |
| Kai Chen, Yupeng Ren, Deborah Gaebler-Spira, and Li-Qun Zhang. “Home-Based Tele-Assisted Robotic Rehabilitation of Joint Impairments in Children with Cerebral Palsy.” 2014 36th Annual International Conference of the IEEE Engineering in Medicine and Biology Society (August 2014). doi:10.1109/embc.2014.6944819. |
| Kassee, Caroline, Carolyn Hunt, Michael W.R. Holmes, and Meghann Lloyd. “Home-Based Nintendo Wii Training to Improve Upper-Limb Function in Children Ages 7 to 12 with Spastic Hemiplegic Cerebral Palsy.” Journal of Pediatric Rehabilitation Medicine 10, no. 2 (June 28, 2017): 145–154. doi:10.3233/prm-170439.* |
| Keller, Jeffrey W., and Hubertus J.A. van Hedel. “Weight-Supported Training of the Upper Extremity in Children with Cerebral Palsy: a Motor Learning Study.” Journal of NeuroEngineering and Rehabilitation 14, no. 1 (August 30, 2017). doi:10.1186/s12984-017-0293-3. |
| Knights, Shannon, Nicholas Graham, Lauren Switzer, Hamilton Hernandez, Zi Ye, Briar Findlay, Wen Yan Xie, Virginia Wright, and Darcy Fehlings. “An Innovative Cycling Exergame to Promote Cardiovascular Fitness in Youth with Cerebral Palsy: A Brief Report.” Developmental Neurorehabilitation (June 20, 2014): 1–6. doi:10.3109/17518423.2014.923056. |
| Levac, Danielle, Anna McCormick, Mindy F. Levin, Marie Brien, Richard Mills, Elka Miller, and Heidi Sveistrup. “Active Video Gaming for Children with Cerebral Palsy: Does a Clinic-Based Virtual Reality Component Offer an Additive Benefit? A Pilot Study.” Physical & Occupational Therapy In Pediatrics 38, no. 1 (April 4, 2017): 74–87. doi:10.1080/01942638.2017.1287810.* |
| Li, W., Lam-Damji, S., Chau, T., & Fehlings, D. (2009). The development of a home-based virtual reality therapy system to promote upper extremity movement for children with hemiplegic cerebral palsy. Technology and Disability, 21(3), 107-113. |
| Liu, Ling, Xiang Chen, Zhiyuan Lu, Shuai Cao, De Wu, and Xu Zhang. “Development of an EMG-ACC-Based Upper Limb Rehabilitation Training System.” IEEE Transactions on Neural Systems and Rehabilitation Engineering 25, no. 3 (March 2017): 244–253. doi:10.1109/tnsre.2016.2560906.* |
| MacIntosh, Alexander, Lauren Switzer, Susan Hwang, Adrian L. Jessup Schneider, Daniel Clarke, T.C. Nicholas Graham, and Darcy L. Fehlings. “Ability-Based Balancing Using the Gross Motor Function Measure in Exergaming for Youth with Cerebral Palsy.” Games for Health Journal 6, no. 6 (December 2017): 379–385. doi:10.1089/g4h.2017.0053.* |
| MacIntosh, Alexander; Switzer, Lauren; Hernandez, Hamilton; Hwang, Susan; Schneider, Adrian L. Jessup; Moran, Daniel; Graham, T. C. Nicholas; Fehlings, Darcy L. “Balancing for Gross Motor Ability Differences in Exercise Videogames Between Youth with Cerebral Palsy at Gross Motor Function Classification System Levels II and III.” Developmental Medicine & Child Neurology 58 (September 2016): 110–111. doi:10.1111/dmcn.50_13225. |
| Meyns, Pieter, Liene Pans, Kaat Plasmans, Lieve Heyrman, Kaat Desloovere, and Guy Molenaers. “The Effect of Additional Virtual Reality Training on Balance in Children with Cerebral Palsy after Lower Limb Surgery: A Feasibility Study.” Games for Health Journal 6, no. 1 (February 2017): 39–48. doi:10.1089/g4h.2016.0069. |
| Nash, Janet, Peter D. Neilson, and Nicholas J. O’Dwyer. “REDUCING SPASTICITY TO CONTROL MUSCLE CONTRACTURE OF CHILDREN WITH CEREBRAL PALSY.” Developmental Medicine & Child Neurology 31, no. 4 (November 12, 2008): 471–480. doi:10.1111/j.1469-8749.1989.tb04025.x. |
| Ni, Lian Ting, Darcy Fehlings, and Elaine Biddiss. “Design and Evaluation of Virtual Reality–Based Therapy Games with Dual Focus on Therapeutic Relevance and User Experience for Children with Cerebral Palsy.” Games for Health Journal 3, no. 3 (June 2014): 162–171. doi:10.1089/g4h.2014.0003. |
| Odle, Brooke M., Amanda Irving, and Richard Foulds. “Usability of an Adaptable Video Game Platform for Children with Cerebral Palsy.” 2009 IEEE 35th Annual Northeast Bioengineering Conference (April 2009). doi:10.1109/nebc.2009.4967748. |
| Pavão, Silvia Leticia, Joice Luiza Bruno Arnoni, Alyne Kalyane Câmara de Oliveira, and Nelci Adriana Cicuto Ferreira Rocha. “Impacto de Intervenção Baseada Em Realidade Virtual Sobre o Desempenho Motor e Equilíbrio de Uma Criança Com Paralisia Cerebral: Estudo de Caso1.” Revista Paulista de Pediatria 32, no. 4 (December 2014): 389–394. doi:10.1016/j.rpped.2014.04.005. |
| Peper, C. (Lieke) E., Edwin C. P. Van Loon, Anke Van de Rijt, Annelie Salverda, and Annette A. van Kuijk. “Bimanual Training for Children with Cerebral Palsy: Exploring the Effects of Lissajous-Based Computer Gaming.” Developmental Neurorehabilitation 16, no. 4 (March 11, 2013): 255–265. doi:10.3109/17518423.2012.760116. |
| Preston, N., A. Weightman, J. Gallagher, M. Levesley, M. Mon-Williams, M. Clarke, and R. J. OConnor. “A pilot single-blind multicentre randomized controlled trial to evaluate the potential benefits of computer-assisted arm rehabilitation gaming technology on the arm function of children with spastic cerebral palsy.” Clinical Rehabilitation 30, no. 10 (September 13, 2015): 1004–1015. doi:10.1177/0269215515604699.* |
| Psychouli, Pavlina, and Colin R. Kennedy. “Modified Constraint-Induced Movement Therapy as a Home-Based Intervention for Children With Cerebral Palsy.” Pediatric Physical Therapy 28, no. 2 (2016): 154–160. doi:10.1097/pep.0000000000000227. |
| Qiu, Qinyin, Diego A Ramirez, Soha Saleh, Gerard G Fluet, Heta D Parikh, Donna Kelly, and Sergei V Adamovich. “The New Jersey Institute of Technology Robot-Assisted Virtual Rehabilitation (NJIT-RAVR) System for Children with Cerebral Palsy: a Feasibility Study.” Journal of NeuroEngineering and Rehabilitation 6, no. 1 (2009): 40. doi:10.1186/1743-0003-6-40. |
| Ramstrand, N., & Lygnegård, F. “Can balance in children with cerebral palsy improve through use of an activity promoting computer game?”. Technology and Health Care, 20 (2012):531-540. doi: 10.3233/THC-2012-0696.* |
| Reifenberg, Grace, Gabrielle Gabrosek, Kelly Tanner, Karen Harpster, Rachel Proffitt, and Andrew Persch. “Feasibility of Pediatric Game-Based Neurorehabilitation Using Telehealth Technologies: A Case Report.” American Journal of Occupational Therapy 71, no. 3 (March 27, 2017): 7103190040p1. doi:10.5014/ajot.2017.024976. |
| Rios, D. C., T. Gilbertson, S. W. McCoy, R. Price, K. Gutman, K. E. F. Miller, A. Fechko, and C. T. Moritz. “NeuroGame Therapy to Improve Wrist Control in Children with Cerebral Palsy: A Case Series.” Developmental Neurorehabilitation 16, no. 6 (April 25, 2013): 398–409. doi:10.3109/17518423.2013.766818. |
| Robert, M., L. Ballaz, R. Hart, and M. Lemay. “Exercise Intensity Levels in Children With Cerebral Palsy While Playing With an Active Video Game Console.” Physical Therapy 93, no. 8 (April 11, 2013): 1084–1091. doi:10.2522/ptj.20120204. |
| Robert, Maxime T., Rhona Guberek, Mindy F. Levin, and Heidi Sveistrup. “Motor Learning of the Upper Limb in Children with Cerebral Palsy after Virtual and Physical Training Intervention.” 2013 International Conference on Virtual Rehabilitation (ICVR) (August 2013). doi:10.1109/icvr.2013.6662125. |
| Robert, Maxime T, and Mindy F Levin. “Validation of Reaching in a Virtual Environment in Typically Developing Children and Children with Mild Unilateral Cerebral Palsy.” Developmental Medicine & Child Neurology 60, no. 4 (February 10, 2018): 382–390. doi:10.1111/dmcn.13688. |
| Rowland, Jennifer L., and James H. Rimmer. “Feasibility of Using Active Video Gaming as a Means for Increasing Energy Expenditure in Three Nonambulatory Young Adults With Disabilities.” PM&R 4, no. 8 (August 2012): 569–573. doi:10.1016/j.pmrj.2012.03.011. |
| Sajan, Jane Elizabeth, Judy Ann John, Pearlin Grace, Sneha Sara Sabu, and George Tharion. “Wii-Based Interactive Video Games as a Supplement to Conventional Therapy for Rehabilitation of Children with Cerebral Palsy: A Pilot, Randomized Controlled Trial.” Developmental Neurorehabilitation 20, no. 6 (November 15, 2016): 361–367. doi:10.1080/17518423.2016.1252970. |
| Sandlund, Marlene, Eva Lindh Waterworth, Suzanne McDonough, and Charlotte Hager Ross. “Interactive Games in Motor Rehabilitation for Children with Sensorimotor Disorders.” 2007 Virtual Rehabilitation (September 2007). doi:10.1109/icvr.2007.4362136. |
| Sandlund, Marlene, Eva Lindh Waterworth, and Charlotte Häger. “Using Motion Interactive Games to Promote Physical Activity and Enhance Motor Performance in Children with Cerebral Palsy.” Developmental Neurorehabilitation 14, no. 1 (January 17, 2011): 15–21. doi:10.3109/17518423.2010.533329.* |
| Sevick, Marisa, Elizabeth Eklund, Allison Mensch, Matthew Foreman, John Standeven, and Jack Engsberg. “Using Free Internet Videogames in Upper Extremity Motor Training for Children with Cerebral Palsy.” Behavioral Sciences 6, no. 2 (June 7, 2016): 10. doi:10.3390/bs6020010.* |
| Sgandurra, Giuseppina, Adriano Ferrari, Giuseppe Cossu, Andrea Guzzetta, Leonardo Fogassi, and Giovanni Cioni. “Randomized Trial of Observation and Execution of Upper Extremity Actions Versus Action Alone in Children With Unilateral Cerebral Palsy.” Neurorehabilitation and Neural Repair 27, no. 9 (July 25, 2013): 808–815. doi:10.1177/1545968313497101. |
| Silva Dias, T., da Conceição, K. F., de Oliveira, A. I. A., & da Silva, R. L. M. The contributions of game therapy concerning motor performance of individual with cerebral palsy. 2017. Brazilian Journal of Occupational Therapy/Cadernos Brasileiros de Terapia Ocupacional, 25(3). |
| Stansfield, S., Dennis, C., Larin, H., & Gallagher, C. Movement-Based VR Gameplay Therapy For A Child With Cerebral Palsy. Annual Review of Cybertherapy and Telemedicine 2015. 2016. Virtual Reality in Healthcare: Medical Simulation and Experiential Interface, 219, 153. |
| Tarakci, Devrim, Burcu Ersoz Huseyinsinoglu, Ela Tarakci, and Arzu Razak Ozdincler. “Effects of Nintendo Wii-Fit®video Games on Balance in Children with Mild Cerebral Palsy.” Pediatrics International 58, no. 10 (August 23, 2016): 1042–1050. doi:10.1111/ped.12942. |
| Van Hedel, Hubertus J.A., Karin Wick, Andreas Meyer-Heim, and Kynan Eng. “Improving Dexterity in Children with Cerebral Palsy.” 2011 International Conference on Virtual Rehabilitation (June 2011). doi:10.1109/icvr.2011.5971872. |
| Velasco, Miguel A., Rafael Raya, Luca Muzzioli, Daniela Morelli, Abraham Otero, Marco Iosa, Febo Cincotti, and Eduardo Rocon. “Evaluation of Cervical Posture Improvement of Children with Cerebral Palsy after Physical Therapy Based on Head Movements and Serious Games.” BioMedical Engineering OnLine 16, no. S1 (August 2017). doi:10.1186/s12938-017-0364-5. |
| Wade, Will, and David Porter. “Sitting Playfully: Does the Use of a Centre of Gravity Computer Game Controller Influence the Sitting Ability of Young People with Cerebral Palsy?” Disability and Rehabilitation: Assistive Technology 7, no. 2 (October 4, 2011): 122–129. doi:10.3109/17483107.2011.589485. |
| Weightman, Andrew Patrick Hayes, Nick Preston, Raymond Holt, Matthew Allsop, Martin Levesley, and Bipinchandra Bhakta. “Engaging Children in Healthcare Technology Design: Developing Rehabilitation Technology for Children with Cerebral Palsy.” Journal of Engineering Design 21, no. 5 (March 25, 2009): 579–600. doi:10.1080/09544820802441092. |
| Winkels, Diny G. M., Anke I. R. Kottink, Rutger A. J. Temmink, Juliëtte M. M. Nijlant, and Jaap H. Buurke. “Wii™-Habilitation of Upper Extremity Function in Children with Cerebral Palsy. An Explorative Study.” Developmental Neurorehabilitation 16, no. 1 (October 3, 2012): 44–51. doi:10.3109/17518423.2012.713401.* |
| Yagüe Sebastián, M.P., M.M. Yagüe Sebastián, A. Lekuona Amiano, and M.C. Sanz Rubio. “Los Videojuegos En El Tratamiento Fisioterápico de La Parálisis Cerebral.” Fisioterapia 38, no. 6 (November 2016): 295–302. doi:10.1016/j.ft.2015.11.005.* |
| Yalon-Chamovitz, Shira, and Patrice L. (Tamar) Weiss. “Virtual Reality as a Leisure Activity for Young Adults with Physical and Intellectual Disabilities.” Research in Developmental Disabilities 29, no. 3 (May 2008): 273–287. doi:10.1016/j.ridd.2007.05.004.* |
| Yong, C. H., Wei, R. F. M., Aimei, M. K., Yanting, P., Shan, C. P., Leng, M. N. Y., & Kumar, D. S. Effects of virtual reality games with physiotherapy on balance of children with cerebral palsy. In Proceedings of the 5th International Conference on Rehabilitation Engineering & Assistive Technology. (2011, July) (p. 54). Singapore Therapeutic, Assistive & Rehabilitative Technologies (START) Centre. |
| Yoo, J. W., Lee, D. R., Sim, Y. J., You, J. H., & Kim, C. J. Effects of innovative virtual reality game and EMG biofeedback on neuromotor control in cerebral palsy. Bio-medical materials and engineering. 2014. 24(6): 3613-3618. doi: 10.3233/BME-141188. |
| Zoccolillo, L., Morelli, D., Cincotti, F., Muzzioli, L., Gobbetti, T., Paolucci, S., & Iosa, M. Video-game based therapy performed by children with cerebral palsy: a cross-over randomized controlled trial and a cross-sectional quantitative measure of physical activity. 2015. Eur J Phys Rehabil Med, 51(6), 669-76. |
| Zondervan, Daniel K., Riccardo Secoli, Aurelia Mclaughlin Darling, John Farris, Jan Furumasu, and David J. Reinkensmeyer. “Design and Evaluation of the Kinect-Wheelchair Interface Controlled (KWIC) Smart Wheelchair for Pediatric Powered Mobility Training.” Assistive Technology 27, no. 3 (May 26, 2015): 183–192. doi:10.1080/10400435.2015.1012607. |
